# Deletion of adipocyte NOS3 potentiates high-fat diet-induced hypertension and vascular remodelling via chemerin

**DOI:** 10.1093/cvr/cvad164

**Published:** 2023-10-28

**Authors:** Andy W C Man, Yawen Zhou, Gisela Reifenberg, Alica Camp, Thomas Münzel, Andreas Daiber, Ning Xia, Huige Li

**Affiliations:** Department of Pharmacology, Johannes Gutenberg University Medical Center, Langenbeckstr. 1, 55131 Mainz, Germany; Department of Pharmacology, Johannes Gutenberg University Medical Center, Langenbeckstr. 1, 55131 Mainz, Germany; Department of Pharmacology, Johannes Gutenberg University Medical Center, Langenbeckstr. 1, 55131 Mainz, Germany; Department of Pharmacology, Johannes Gutenberg University Medical Center, Langenbeckstr. 1, 55131 Mainz, Germany; Department of Cardiology, Cardiology I, Johannes Gutenberg University Medical Center, Mainz, Germany; German Centre for Cardiovascular Research (DZHK), Partner Site Rhine-Main, Mainz, Germany; Department of Cardiology, Cardiology I, Johannes Gutenberg University Medical Center, Mainz, Germany; German Centre for Cardiovascular Research (DZHK), Partner Site Rhine-Main, Mainz, Germany; Department of Pharmacology, Johannes Gutenberg University Medical Center, Langenbeckstr. 1, 55131 Mainz, Germany; Department of Pharmacology, Johannes Gutenberg University Medical Center, Langenbeckstr. 1, 55131 Mainz, Germany; German Centre for Cardiovascular Research (DZHK), Partner Site Rhine-Main, Mainz, Germany

**Keywords:** Oxidative stress, Endothelial function, Adipokine, Adipose tissue, Cross-talk

## Abstract

**Aims:**

Obesity is an epidemic that is a critical contributor to hypertension and other cardiovascular diseases. Current paradigms suggest that endothelial nitric oxide synthase (eNOS/NOS3) in the vessel wall is the primary regulator of vascular function and blood pressure. However, recent studies have revealed the presence of eNOS/NOS3 in the adipocytes of white adipose tissues and perivascular adipose tissues (PVATs). The current understanding of the role of adipocyte NOS3 is based mainly on studies using global knockout models. The present study aimed to elucidate the functional significance of adipocyte NOS3 for vascular function and blood pressure control.

**Methods and results:**

We generated an adipocyte-specific NOS3 knockout mouse line using adiponectin promoter-specific Cre-induced gene inactivation. Control and adipocyte-specific NOS3 knockout (A-NOS3 KO) mice were fed a high-fat diet (HFD). Despite less weight gain, A-NOS3 KO mice exhibited a significant increase in blood pressure after HFD feeding, associated with exacerbated vascular dysfunction and remodelling. A-NOS3 KO mice also showed increased expression of signature markers of inflammation and hypoxia in the PVATs. Among the differentially expressed adipokines, we have observed an upregulation of a novel adipokine, chemerin, in A-NOS3 KO mice. Chemerin was recently reported to link obesity and vascular dysfunction. Treatment with chemerin neutralizing antibody normalized the expression of remodelling markers in the aorta segments cultured in serum from HFD-fed A-NOS3 KO mice *ex vivo*.

**Conclusion:**

These data suggest that NOS3 in adipocytes is vital in maintaining vascular homeostasis; dysfunction of adipocyte NOS3 contributes to obesity-induced vascular remodelling and hypertension.

## Graphical Abstract

**Graphical Abstract:**
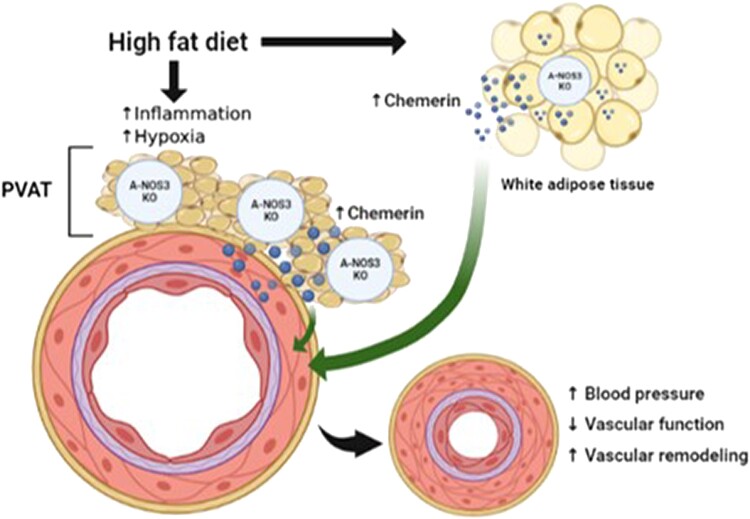

**Time of primary review: 26 days**

## 1. Introduction

Obesity is an epidemic that affects over one-third of the world’s population nowadays^1^ and, by estimation, up to 57.8% of the world's adult population could be either overweight or obese by 2030.^2^ Obesity is a multifactorial and largely preventable disease influenced by environmental, socioeconomic, and genetic factors.^3^ It is associated with an increased risk of nearly every chronic health complication, including diabetes, kidney disease, stroke, hypertension, and other cardiovascular diseases.^3,4^ Among these factors, obesity is a critical contributor to hypertension in humans.^5^ Despite numerous studies, the underlying mechanisms through which excessive fat accumulation and/or obesity result in an increased incidence of hypertension are still controversial.

In obesity, endothelial dysfunction, the phenotypic switch to a pro-atherosclerotic phenotype, is one of the earliest vascular alterations observed.^6^ Obesity-induced endothelial dysfunction exhibits a reduced bioavailability of nitric oxide (NO)^7^ and a predominant generation of endothelium-derived contracting factors in the endothelium.^6^ In obese patients, endothelial dysfunction has been observed along with vascular inflammation and oxidative stress.^8^ In addition, obesity, vascular inflammation, and oxidative stress are often associated with vascular remodelling, characterized by smooth muscle proliferation and arterial stiffness in both animal models and human studies.^9,10^ Nevertheless, the complexity of the underlying mechanisms involved in vascular alterations during obesity requires further exploration.

Endothelial nitric oxide synthase (eNOS; also known as NOS3 or NOSIII) is named after the cell type (endothelial cell) in which it was first identified. eNOS is an enzyme well-known for its role in generating vasoprotective NO. Numerous studies using global eNOS-deficient mice have demonstrated the anti-hypertensive, anti-thrombotic, and anti-atherosclerotic effects of eNOS, mainly attributed to the NO derived from the endothelium.^11–14^

Recently, eNOS expression has been found in the adipocytes of various adipose tissues, contributing to the production of vascular NO and modulating vascular (patho)physiology.^15,16^ Among different adipose tissues, perivascular adipose tissue (PVAT) plays a crucial role in obesity-induced vascular dysfunction.^17,18^ In healthy conditions, PVAT has anti-contractile properties and modulates the normal function of the vasculature.^17,19^ In obesity, adipose tissues become inflamed and dysfunctional, while inflammation can cause the phenotypic switch of PVAT from anti-inflammatory to pro-inflammatory.^20^ Several studies using high-fat diet (HFD) and/or genetic manipulation models have suggested the pathophysiological significance of adipose tissue eNOS in mediating vascular functions, inflammation, and other metabolic processes.^16,21,22^

Nevertheless, while most of these studies relied mainly on the use of eNOS global knockout mice or pharmacological inhibition of NOS isoforms, no studies have provided direct evidence using a model with specific NOS3 (eNOS) knockout in the adipocyte. Therefore, we generated a tamoxifen-induced adipocyte-specific NOS3 knockout mouse model (A-NOS3 KO) to investigate the function of adipocyte NOS3 in diet-induced obesity directly. In the present study, we examined the vascular function and phenotype in this animal model. We found that adipocyte-specific NOS3 knockout mice exhibited exacerbated obesity-induced hypertension associated with endothelial dysfunction and vascular remodelling. Strikingly, the expression of a novel adipokine that links obesity and vascular dysfunction, chemerin,^23^ was remarkably increased in A-NOS3 KO mice. These data indicate that adipose NOS3 may play an essential role in modulating vascular function, at least partly, via chemerin.

## 2. Methods

### 2.1 Adipocyte NOS3 knockout mice

**Figure 1:**
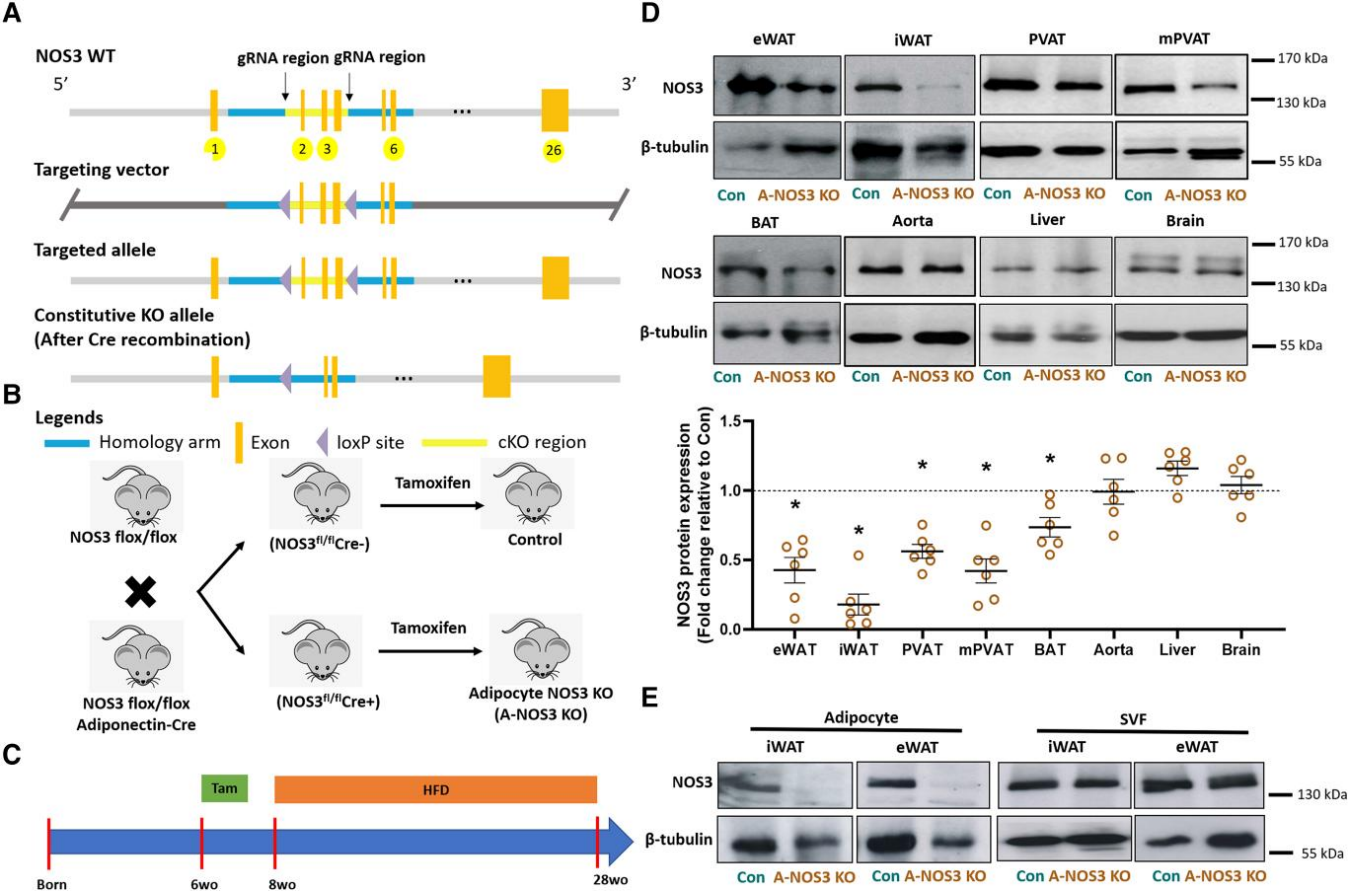
Generation of adipocyte-specific NOS3 KO mice and experimental settings. (*A*) Gene-targeting strategy with the position of loxP sites inserted within the gene-targeting construct before and after exon 2 to 4 of Nos3 was used to generate the founder NOS3^flox/flox^ mice. (*B*) To obtain adipocyte NOS3 knockout mice and their respective control, the founder NOS3^flox/flox^ mice were crossed with adipocyte-specific tamoxifen-inducible Adipoq-iCreERT2 mice to obtain either NOS3^flox/flox^Adipoq-Cre^+^ or NOS3^flox/flox^Adipoq-Cre^−^ mice. To induce adiponectin promoter-specific activation of the Cre-recombinase, tamoxifen (2 mg/mouse/day) were injected intraperitoneally in both NOS3^flox/flox^Adipoq-Cre^+^ (A-NOS3 KO) and NOS3^flox/flox^Adipoq-Cre^−^ (control) mice for 5 consecutive days at the age of 6 weeks. These mice were allowed a 7-day waiting period after the last injection. (*C*) Starting at the age of 8 weeks, control and A-NOS3 KO mice were randomly assigned to be fed with either NCD or HFD (45% kcal from fat) and freely assess to drinking water. The HFD treatment period was 20 weeks. (*D*) Western blot showing the protein expression of NOS3 in the different tissues, including epididymal and inguinal white adipose tissues (eWAT and iWAT), aortic PVAT, mesenteric perivascular adipose tissue (mPVAT), BAT, aorta, liver, and brain. Results of western blots were quantified to show the relative protein expression of NOS3 in A-NOS3 KO mice compared to that of control. Student’s *t*-test was used to compare control and A-NOS3 KO groups. *P < 0.05 vs. control. (*E*) Western blot showing the protein expression of NOS3 in the mature adipocytes and SVF isolated from iWAT and eWAT from control and A-NOS3 KO mice.

The NOS3flox/flox mice on a C57BL/6J genetic background were generated at Cyagen Biosciences (Cyagen US Inc., Santa Clara, CA, USA). NOS3 null mutation was made using clustered regularly interspaced short palindromic repeats/Cas9-mediated genome engineering technique. Exons 2–4 with 1384 bp nucleotide from NOS3 gene were deleted, resulting in a frameshift. Adipoq-iCreERT2 mice were purchased from The Jackson Laboratory (Stock 02512425124). This mouse strain enables tamoxifen-inducible Cre-mediated recombination in adipocytes.^24^ Homozygous NOS3flox/flox mice were crossed with Adipoq-iCreERT2 mice to obtain NOS3flox/floxAdipoq-Cre^+^ mice. Then, NOS3flox/flox mice were crossed with NOS3flox/floxAdipoq-Cre^+^ mice to obtain either NOS3flox/floxAdipoq-Cre^+^ or NOS3flox/floxAdipoq-Cre^−^ mice for experiments. To induce adiponectin promoter-specific activation of the Cre-recombinase, tamoxifen (2 mg/mouse/day) was injected intraperitoneally in both NOS3flox/floxAdipoq-Cre^+^ (A-NOS3 KO) and NOS3flox/floxAdipoq-Cre^−^ (control) mice for 5 consecutive days at the age of 6 weeks (*Figure 1*). These mice were allowed a 7-day waiting period after the last injection.^24^ The animal experiment was approved by the responsible regulatory authority (Landesuntersuchungsamt Rheinland-Pfalz; 23 177-07/G 17-1-020 and G 22-1-039) and was conducted in accordance with the German animal protection law and the National Institutes of Health (NIH) Guide for the Care and Use of Laboratory Animals. The animals were kept in adequate groups and provided with nesting material and enrichment. At the end of the experiment, mice were anaesthetized with isoflurane and euthanized by an overdose of pentobarbital (150 mg/kg body weight) injected intraperitoneally.

### 2.2 HFD treatment

Starting at the age of 8 weeks, control and A-NOS3 KO mice were randomly assigned to be fed with either normal chow diet (NCD) or a HFD (45% kcal from fat; E15744-34, ssniff®, Soest, Germany) and freely assess to drinking water. The HFD treatment period was 20 weeks (*Figure 1*).^16,25^

### 2.3 Blood pressure measurement

Blood pressure parameters were measured noninvasively in conscious mice by a computerized system (CODA Monitor, Kent Scientific, Torrington, CT, USA) as described in our previous studies.^26–29^ Briefly, the occlusion cuff and the volume-pressure recording cuff were placed close to the base of the tail. After an adaptation period of 30 min on a 37°C warm pad, 10 preliminary measurements were performed before the actual measurement. Mice were acclimated for three consecutive days before the actual measurement. Results are presented as the mean of at least 15 recordings on each occasion. The measurements were performed at the same time of a day from 2 pm to 4 pm by the same investigator.

### 2.4 Isometric tension studies

The vascular function of the aortas and second-order mesenteric arteries was accessed by wire myograph as described in our previous studies.^27^ Vessels were either dissected free of adherent connective tissues (without PVAT) or were left PVAT intact (with PVAT) and placed in cold modified Krebs-Ringer bicarbonate buffer under continuous aeration with 95% oxygen (O_2_)/5% carbon dioxide (CO_2_). Vessel rings (2–3 mm long) were suspended in the chambers of a Mulvany–Halpern wire myograph system (620 M, Danish Myo Technology A/S, Aarhus, Denmark). The isometric force was recorded by a PowerLab/8SP system (AD Instruments Inc., Colorado Springs, CO, USA). The preparations were equilibrated for 30 min at the optimal resting tension. The viability of the endothelium was tested by the relaxation response to a single dose of acetylcholine (10^−4^ M) after obtaining a reference contraction to 60 mM potassium chloride (KCl) twice before the actual experiment. Preparations were then pre-contracted by exposing them to increasing concentrations of phenylephrine (PE, 10^−9^ to 10^−5^ M). Endothelium-dependent relaxation was examined by exposure to increasing concentrations of acetylcholine (10^−9^ to 10^−4^ M). Change in tension is expressed as a percentage of the PE contraction (∼80% of the reference KCl contraction). The area under the relaxation curve (AURC) was measured in dose-dependent curves of the preparation with or without PVAT. The difference (ΔAURC) between AURC of the curve with PVAT and without PVAT was calculated. The anti-contractile effect of PVAT was presented as the ΔAURC with reference to the control group for comparison. The compounds used were purchased from Sigma-Aldrich, Taufkirchen, Germany.

### 2.5 Adipocytes and stromal vascular fraction separation

Adipose tissues were isolated from 12-week-old mice. Adipose tissues were minced into tiny pieces and incubated in 5 mL of digestion buffer (2 mg/mL Collagenase I, 0.5% bovine serum albumin, 10 mM CaCl_2_, in phosphate-buffered saline [PBS]) at 37°C 30 min with gentle agitation. After incubation, the digestion was stopped by adding 5 mL of basal medium [Dulbecco's modified Eagle's medium (DMEM) with 10% fetal bovine serum (FBS) and 1% Pen/Strep] to the homogenate. The homogenate was filtered through a 70 μm cell strainer and centrifuged at 800 g at 4°C for 15 min to separate the cells into layers. After centrifugation, the white floating top layer above the supernatant was concentrated with mature adipocytes. In contrast, the stromal vascular fraction (SVF) was concentrated as a brownish pellet at the bottom of the tube. The mature adipocytes and SVF were collected into individual tubes, washed twice with PBS, and used for further experiments.

### 2.6 Adipocyte differentiation

SVF from the control mice and A-NOS3 KO mice were cultured in 10 mL of complete medium (DMEM containing 10% FBS and 1% Pen/Strep) until 70–80% confluence. The cells were then trypsinized, plated on collagen-coated dished, and incubated in complete medium for 1–2 h. Then, the cells were washed with PBS twice to remove red blood cells, immune cells, and other contaminants, and cultured in the complete medium. After reaching 95% confluence, the cells were incubated with induction medium (complete medium + 5 μg/mL insulin, 1 nM T3, 125 μM indomethacin, 2 μg/mL dexamethasone, 0.5 μM 3-isobutyl-1-methylxanthine and 0.5 μM rosiglitazone) for 48 h. After 2 days of induction, the cells were incubated with maintenance medium (complete medium + 5 μg/mL insulin, 1 nM T3 and 0.5 μM rosiglitazone) for another 48 h. After 2 days, the cells were incubated with maintenance medium (complete medium + 5 μg/mL insulin, 1 nM T3, and 1 μM rosiglitazone) for another 3 days. After that, cells were fully differentiated to mature fat cells and filled with oil droplets. The cells were then used for western blot and quantitative PCR (qPCR) experiments. The reagents used in adipocyte isolation and differentiation were purchased from Sigma-Aldrich.

### 2.7 Gene expression studies by qPCR

Total RNA was isolated using peqGOLD TriFast™ (PEQLAB, Erlangen, Germany) and cDNA was reverse transcribed using High Capacity cDNA Reverse Transcription Kit (Applied Biosystems, Waltham, MA, USA) according to our previous publication.^25^ QPCR was performed using SYBR Green JumpStart^TM^*Taq* Ready-Mix^TM^ (Sigma-Aldrich) on an iCycler Real-Time PCR Detection System (Bio-Rad, Waltham, MA, USA). Quantification was achieved by the difference in quantification cycles (ΔΔCt) values that were normalized to reference genes (β-actin). Relative gene expression of the target gene in each sample was expressed as the percentage of control. The specificity of the qPCR primers was checked by melting curve analysis or gel electrophoresis of the qPCR product. The sequences of the primers used are listed in Supplementary material online, *Table S1*.

### 2.8 Protein expression by western blotting

Aorta and adipose tissue samples were homogenized in radioimmunoprecipitation assay buffer with 1% (v/v) proteinase inhibitor cocktail (#78442, Thermo Fisher Scientific, Waltham, MA, USA). Same amount of lysate protein was loaded and separated in sodium dodecyl sulfate-polyacrylamide gel electrophoresis (SDS-PAGE). The resolved proteins were transferred onto nitrocellulose membranes and probed with specific primary antibody at 4°C overnight with agitation. Membranes were cut and probed with either specific antibody of interest or with antibodies recognizing glyceraldehyde 3-phosphate dehydrogenase (GAPDH) or β-tubulin (as loading control). The protein bands were visualized using enhanced chemiluminescence reagents (GE Healthcare, Chicago, IL, USA) and exposed to Fujitsu Biomedical film (Fujitsu, Tokyo, Japan). Quantification protein expression was based on the ratio of the target protein to either β-tubulin or GAPDH. The following primary antibodies were used: anti-chemerin (#AF2325, Bio-Techne GmbH, Wiesbaden, Germany, 1:1000), anti-p-eNOS (Ser1177) (#9571S, Cell Signaling, Danvers, MA, USA, 1:1000), anti-eNOS (#610297, BD, Franklin Lakes, NJ, USA, 1:1000), anti-p-VASP (Ser239) (#3114S, New England Biolabs, Ipswich, MA, USA, 1:1000), anti-VASP (#3132S, New England Biolabs, 1:1000), anti-β-tubulin (#T7816, Sigma-Aldrich, 1:30000), and anti-GAPDH (#2251-1, Epitomics, Burlingame, CA, USA, 1:30000).^28,29^

### 2.9 Masson’s trichome staining

Aorta samples were fixed in 4% paraformaldehyde and embedded in paraffin. Microtome sectioning was performed to obtain slides with a thickness of 5 µm. After deparaffination and rehydration, Trichrome Stain Kit (Abcam, Cambridge, UK) was used to analyse collagen fibres according to the manufacturer’s instructions. In brief, the slides were incubated in the pre-heated Bouin’s solution at 60°C for 1 h. The yellow colour on the slides was removed by rinsing in running tap water, followed by staining in working Weigert’s iron haematoxylin solution. The slides were then rinsed in deionized water and stained in working phosphomolybdic/phosphotungstic acid solution, followed by aniline blue solution and 1% acetic acid. The slides were then rinsed, dehydrated, and mounted. The collagen staining per image was quantified by NIH ImageJ software. In brief, the images were separated in red-green-blue stacks, and the pixel intensity of the blue colour was quantified using the same threshold among images. Staining was performed in samples from six animals in each group.^28,29^

### 2.10 Immunohistochemistry staining

Aorta samples were fixed in 4% paraformaldehyde and embedded in paraffin. Microtome sectioning was performed to obtain slides with a thickness of 5 µm. After deparaffination and rehydration, slides were immersed in warm EnVision FLEX target Retrieval Solution (#K8004, Agilent, Santa Clara, CA, USA) for antigen retrieval. The slides were then immersed in peroxidase blocking solution to inhibit peroxidase activity. The sections were incubated with primary antibody of Ki-67 (#ab16667, Abcam, 1:50) in 4°C overnight. After washing twice in phosphate buffered saline with 0.1% Tween 20 (PBS-T), the sections were incubated with horseradish peroxidase (HRP)-linked anti-rabbit antibody (#K4002, Agilent, USA) at room temperature for 1 h. After washing twice in PBS-T, the sections were incubated with 3,3'-diaminobenzidine (DAB) staining kit (#K3468, Agilent, USA) for 5 min at room temperature. The slides were rinsed in distilled water, followed by dehydration and mounting. The positive staining per image was quantified by NIH ImageJ software. Images were adjusted to 8-bits and the pixel intensity was quantified using the same threshold among images.^29^

### 2.11 Enzyme-linked immunosorbent assay

Serum levels of mouse chemerin were examined using enzyme-linked immunosorbent assay (ELISA) according to the manufacturer’s instruction (#DY2325, Bio-Techne, USA). In brief, 100 µL diluted serum (1:500 dilution) was added as samples. Samples and standards were then incubated at room temperature for 120 min with gentle shaking. After washing three times, detection antibodies were added and incubated at room temperature for another 120 min. After washing three times, Streptavidin-HRP was added and set for 20 min at room temperature with gentle shaking. After washing five times, the substrate reagent was added in the dark with gentle shaking. After 20 min of incubation, stop solution was added, and the absorption was read at 450 nm with wavelength correction at 570 nm immediately with Sunrise^TM^ microplate reader (Tecan Group, Männedorf, Switzerland) and analysed by Magellan^TM^ software (Tecan Group, Switzerland).^30^

### 2.12 *Ex vivo* aorta culture

Aorta from 12 weeks old control mice fed with NCD (donor mice) was isolated and PVAT was removed from the aorta. Aorta was cut into small rings (∼2 mm long) and the aorta segments were cultured in basal DMEM (Sigma) in the presence of 15% serum of either the donor mice itself, serum of control HFD group or A-NOS3 KO HFD group. Some other aorta segments were incubated additionally with either goat IgG (10 µg/mL) or chemerin neutralizing antibody (10 µg/mL, #AF2325, Bio-Techne, USA). The aorta segments were incubated for 48 h. After that, the aorta segments were collected for RNA extraction and qPCR experiments, as mentioned above. qPCR results of this *ex vivo* aorta culture experiment were normalized against the group cultured with the serum of donor mice.

### 2.13 Statistics data and analysis

Statistical analysis was undertaken only for studies where each group size was at least *n* = 5. *N*-values indicate the number of animals analysed in each group. Results were expressed as mean ± SEM (standard error of the mean). Each individual sample was presented as points in the bar charts, or otherwise, the *N* number was stated in the figure legends. mRNA expression data were normalized to control or baseline to show the relative changes. Student’s *t*-test was used to compare two groups, and one-way analysis of variance (ANOVA) followed by Tukey's *post hoc* test was used to compare multiple groups. *P* values < 0.05 were considered significant. The data analyses were blinded. GraphPad Prism 9.0.1 (GraphPad Software, La Jolla, CA, USA) was used to generate graphs and statistical analysis.

## 3. Results

### 3.1 Generation of adipocyte-specific NOS3 KO mice and experimental settings

In collaboration with Cyagen, we designed and obtained a founder line carrying a floxed NOS3 with loxP site insertion between exon2–4 for Cre-mediated excision (*Figure 1A*). The eNOS^flox/flox^ mice were crossed with tamoxifen-inducible adipocyte-specific Cre-expressing mice (Adipoq-iCreERT2 mice) to generate either NOS3^flox/flox^Adipoq-Cre^+^ (A-NOS3 KO) or NOS3^flox/flox^Adipoq-Cre^−^ (Control) mice for experiments. Once activated by tamoxifen, the Cre-recombinase expressed in NOS3^flox/flox^Adipoq-Cre^+^ mice removed the floxed segment and this Cre-induced cleavage of exon2–4 led to the frameshift and knockout of NOS3 specifically in adipocytes. In control (NOS3^flox/flox^Adipoq-Cre^−^) littermate mice lacking the Cre-recombinase, tamoxifen does not lead to genetic recombination (*Figure 1B*). For the experiments, tamoxifen was intraperitoneally injected to the mice for 5 consecutive days at the age of 6 weeks and HFD was given at the age of 8 weeks for 20 weeks (*Figure 1C*). NOS3 protein expressions in different tissues were examined by western blots, and the result showed a significant reduction of NOS3 expression in the white adipose tissues, brown adipose tissue (BAT), and PVATs, but not in other non-targeted organs (*Figure 1D*). In addition, mature adipocytes and the SVF isolated from white adipose tissues were examined by western blots, confirming the adipocyte-specific knockout of NOS3 in the A-NOS3 KO mice (*Figure 1E*).

### 3.2 Adipocyte-specific NOS3 KO potentiates HFD-induced hypertension despite less weight gain

**Figure 2:**
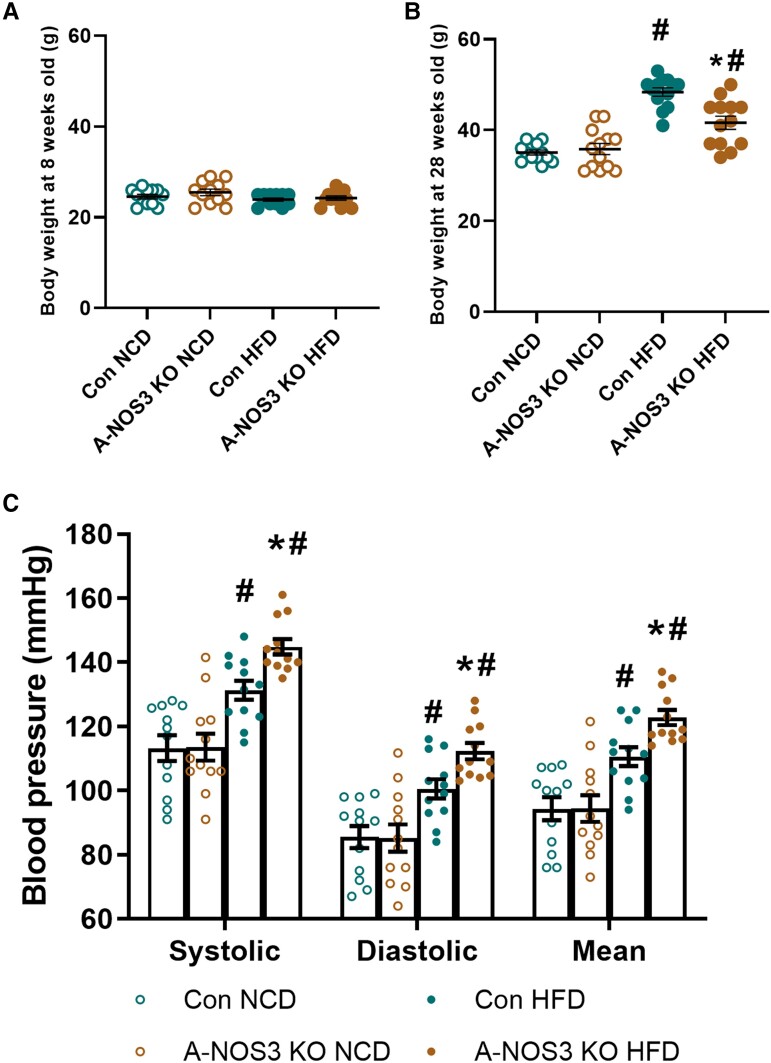
Adipocyte-specific NOS3 KO potentiates HFD-induced hypertension despite less weight gain. (*A*) Body weight of control and A-NOS3 KO mice measured at 8 weeks old (before the experiment). (*B*) Body weight of control and A-NOS3 KO mice fed with either NCD or HFD measured at the age of 28 weeks (after 20 weeks of NCD/HFD feeding). (*C*) Systolic, diastolic and mean arterial blood pressure of mice at the age of 28 weeks was measured by the non-invasive tail-cuff method. One-way ANOVA followed by Tukey's *post hoc* test was used to compare multiple groups. *P < 0.05 vs. control of the same diet. ^#^*P* < 0.05 vs. NCD of the same genotype.

At first, mice were randomly assigned to feed with either NCD or HFD. The body weight of the four groups of mice has no significant difference at the age of 8 weeks before NCD/HFD feeding (*Figure 2A*). At the age of 28 weeks (i.e. after 20 weeks of HFD feeding), both the control HFD and A-NOS3 KO HFD group had significantly increased body weight compared to their NCD groups. Surprisingly, the A-NOS3 KO HFD group had significantly lower body weight than the control HFD group (*Figure 2B*). Under NCD feeding, there were no significant differences in the systolic, diastolic, and mean blood pressure between the control and A-NOS3 groups. Control HFD groups had significantly higher blood pressure than the control NCD group. HFD feeding led to a much larger blood pressure increase in A-NOS3 KO group. The systolic blood pressure of A-NOS3 KO HFD group was ∼14 mmHg higher than that of the control HFD group (*Figure 2C*).

### 3.3 Vascular functions are compromised in adipocyte-specific NOS3 KO mice

**Figure 3:**
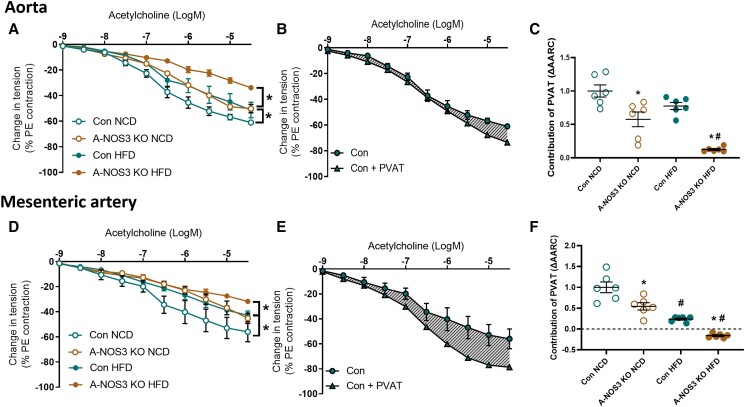
Vascular functions are compromised in adipocyte-specific NOS3 KO mice. The vascular responsiveness of the aorta and second-order mesenteric artery of the mice was studied using wire myography. Preparations were pre-contracted with PE. Basal endothelium-dependent relaxation was assessed by exposing the preparations to increasing concentrations of acetylcholine. (*A*) Endothelium-dependent relaxation in the isolated aortas (without PVAT) from control NCD, A-NOS3 KO NCD, control HFD, and A-NOS3 KO HFD mice. *n* = 6. (*B*) Schematic showing of the contribution of PVAT to acetylcholine-induced relaxation by calculating the area between the relaxation curves of aorta samples with or without surrounding PVAT. (*C*) The contribution of PVAT in endothelium-dependent relaxation was calculated and compared with control NCD as reference. (*D*) Endothelium-dependent relaxation in the isolated mesenteric arteries (without mesenteric PVAT) from control NCD, A-NOS3 KO NCD, control HFD, and A-NOS3 KO HFD mice. *n* = 6. (*E*) Schematic showing of the contribution of mesenteric PVAT (mPVAT) to acetylcholine-induced relaxation by calculating the area between the relaxation curves of mesenteric arteries with or without surrounding mPVAT. (*F*) The contribution of mPVAT in endothelium-dependent relaxation was calculated and compared with control NCD as reference. One-way ANOVA followed by Tukey's *post hoc* test was used to compare multiple groups. *P < 0.05 vs. control of the same diet. ^#^*P* < 0.05 vs. NCD of the same genotype.

In the aorta, there was a significant difference in the basal endothelium-dependent vasodilatory response to acetylcholine between control NCD and A-NOS3 KO NCD groups. At the same time, A-NOS3 KO NCD had a reduced vasodilatory response to acetylcholine compared to control NCD (*Figure 3A*). Both HFD groups showed diminished vasodilatory response to acetylcholine compared to their respective NCD groups, and A-NOS3 KO HFD had a significantly reduced vasodilatory response to acetylcholine compared to control HFD (*Figure 3A*). Next, we investigated the vasodilatory response to acetylcholine in aortas with or without the surrounding PVAT. The contribution of PVAT in the vasodilatory response was calculated by the area between the relaxation curves with or without PVAT (*Figure 3B*) with that of the control NCD group as a reference. The contribution of aortic PVAT in the vasodilatory response was reduced in A-NOS3 KO NCD group compared to the control NCD. HFD feeding has significantly abated the contribution of PVAT in the vasodilatory response in A-NOS3 KO HFD group compared to its NCD group (*Figure 3C*).

Next, we investigated the vascular function in the second-order mesenteric arteries. The endothelium-dependent vasodilatory response to acetylcholine was reduced in A-NOS3 KO NCD group compared to control NCD (*Figure 3D*). Both HFD groups had reduced vasodilatory response to acetylcholine compared to their respective NCD groups, and A-NOS3 KO HFD mice had a significantly reduced vasodilatory response to acetylcholine compared to control HFD mice (*Figure 3D*). The contribution of mesenteric PVAT (mPVAT) in the vasodilatory response was calculated by the area between the relaxation curves with or without mPVAT (*Figure 3E*). The contribution of mPVAT in the vasodilatory response was reduced in A-NOS3 KO NCD group compared to the control NCD group. HFD feeding significantly reduced the PVAT function in both the control and A-NOS3 KO groups. A-NOS3 KO HFD groups even had a negative value in the contribution of mPVAT in the vasodilatory response, suggesting an exertion of contraction effect by the mPVAT due to its dysfunction in the A-NOS3 KO HFD group (*Figure 3F*).

### 3.4 Adipocyte-specific NOS3 KO leads to vascular remodelling

**Figure 4:**
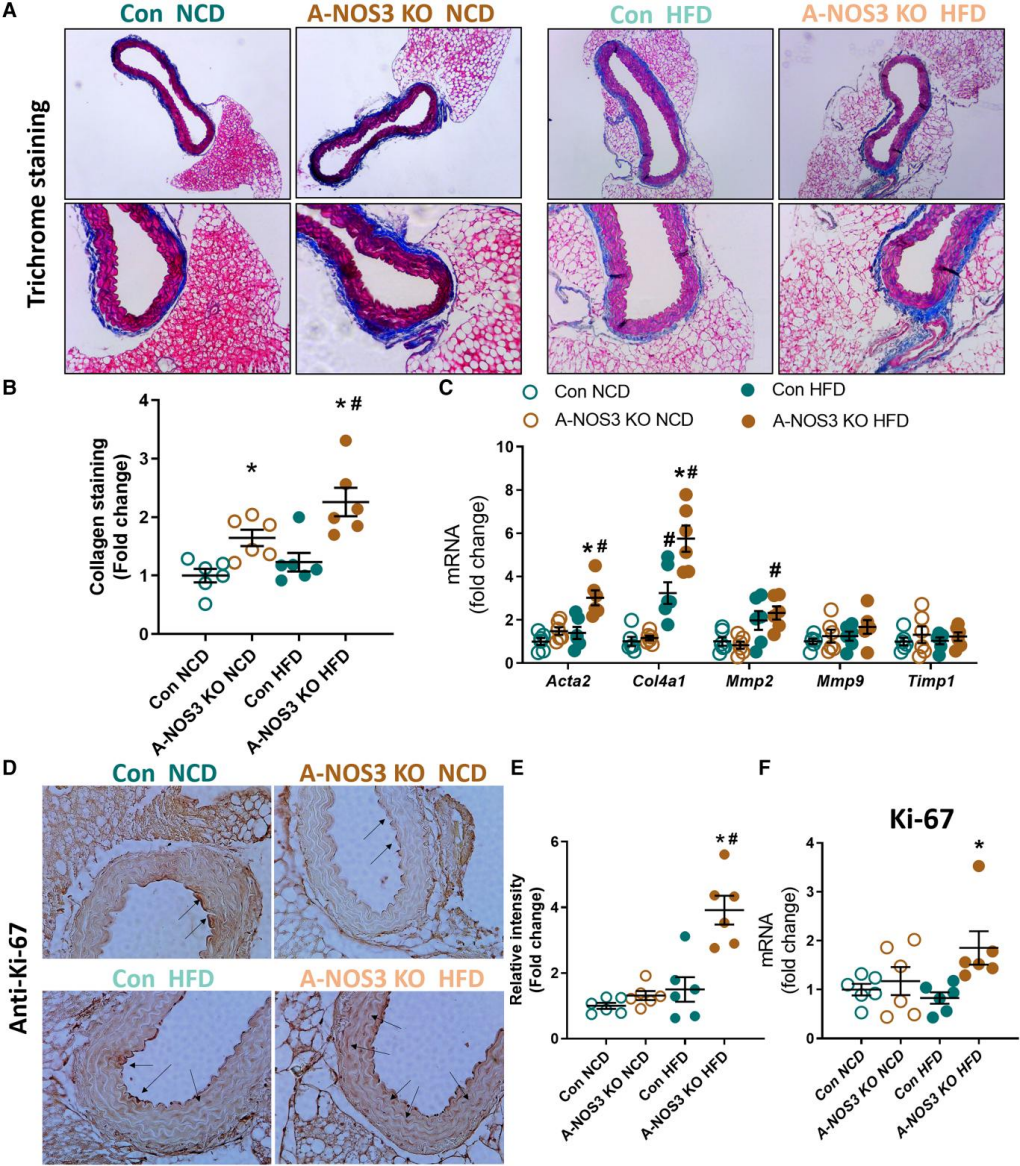
Adipocyte-specific NOS3 KO leads to vascular remodelling. (*A*) Masson’s Trichome staining of aorta sections. Blue indicates the area of collagen deposition. (*B*) Collagen staining from independent samples was quantified by the pixel intensity using ImageJ software and calculated with reference to that of control NCD group. (*C*) The expression of remodelling genes, including smooth muscle alpha actin (*Acta2*), collagen type IV alpha 1 chain (*Col4a1*), matrix metalloproteinase 2 and 9 (*Mmp2* and *Mmp9*) and metallopeptidase inhibitor 1 (*Timp1*) in the aorta samples was analysed by quantitative PCR. (*D*) The expression of proliferation marker Ki-67 was examined by immunohistochemistry staining using anti-Ki-67 antibody. Magnification: 400×. Black arrows indicate areas with positive stain. (*E*) Quantification of the pixel intensity of Ki-67 was performed using ImageJ software calculated with reference to that of control NCD group. (*F*) The gene expression of Ki-67 in the aorta samples was analysed by quantitative PCR. One-way ANOVA followed by Tukey's *post hoc* test was used to compare multiple groups. *P < 0.05 vs. control of the same diet. ^#^*P* < 0.05 vs. NCD of the same genotype.

Next, we investigated the collagen deposition in the aortic wall by Masson’s Trichome staining (*Figure 4A*). Collagen deposition was stained blue, and the area of blue colour was quantified. A-NOS3 KO NCD mice had increased collagen deposition in the aorta compared to the control NCD mice (*Figure 4B*). HFD feeding did not increase collagen deposition in the control group, while A-NOS3 KO HFD group had significantly higher collagen staining level compared to A-NOS3 KO NCD and control HFD groups (*Figure 4B*). In addition, more pronounced blue staining (collagen) was found in the intimal layer in the aorta of A-NOS3 KO HFD group. Coherent with the histological staining, qPCR results showed that the expression levels of collagen metabolism-related genes including smooth muscle alpha actin (*Acta2*), collagen type IV alpha 1 chain (*Col4a1*) and matrix metallopeptidase 2 (*Mmp2*) were significantly upregulated in the aorta of A-NOS3 HFD mice (*Figure 4C*).

We have also examined the vascular smooth muscle proliferation in the aorta. Immunohistological staining using anti-Ki-67 antibody showed that there were more Ki-67-positive cells in the aortic wall in A-NOS3 KO HFD mice compared to control HFD, while there was no significant difference between the control NCD and A-NOS3 KO NCD group (*Figure 4D* and *E*). In addition, the gene expression of Ki-67 was significantly upregulated in A-NOS3 KO HFD mice compared to control HFD (*Figure 4F*). Moreover, the expression of genes related to oxidative stress, including nicotinamide adenine dinucleotide phosphate (NADPH) oxidase 2 (*Nox2*), *p22phox*, *p47phox* was significantly upregulated in A-NOS3 KO HFD mice compared to control HFD (see Supplementary material online, *Figure S1A*). The protein expression of NOS3 in the aorta (without PVAT) was not significantly changed. In these PVAT-free aorta samples, there was no significant difference in the vasodilator-stimulated phosphoprotein (VASP) phosphorylation level between control and A-NOS3 KO mice under basal conditions without NOS3 stimulation, either (see Supplementary material online, *Figure S1B*).

### 3.5 Inflammation and hypoxia are augmented in HFD-fed adipocyte-specific NOS3 KO mice

**Figure 5:**
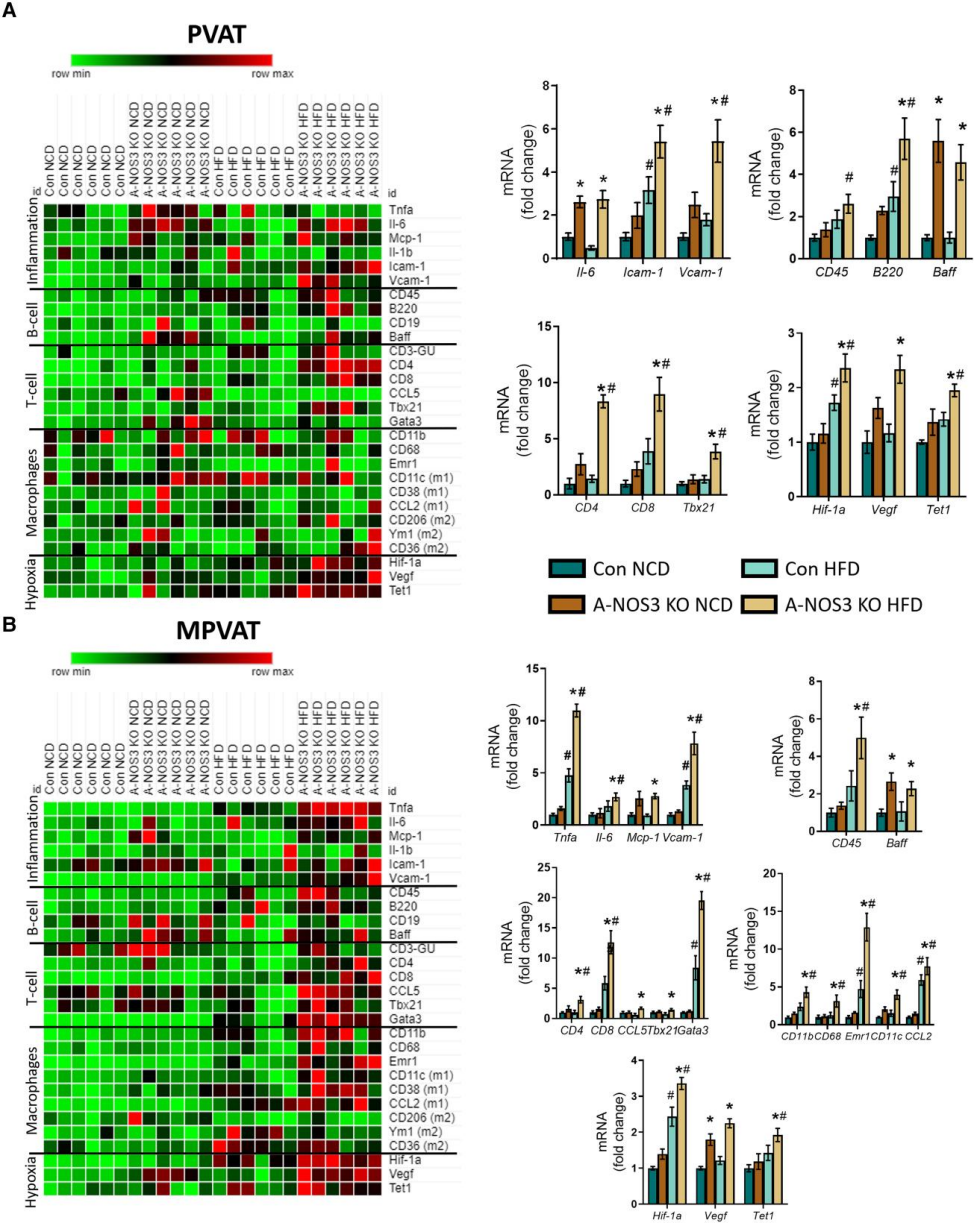
Inflammation and hypoxia are augmented in HFD-fed adipocyte-specific NOS3 KO mice. qPCR heatmap of relative expression of 28 genes comparing control and A-NOS3 KO mice under NCD and HFD in PVAT (*A*) and mPVAT (*B*). These genes include markers of inflammation, immune cells and hypoxia. Red indicates high expression and green indicates low expression relatively. Bar charts in the right panels show the relative expression of the discussed genes. One-way ANOVA followed by Tukey's *post hoc* test was used to compare multiple groups. Quantitative values are shown in Supplementary material online, *Figure S3*.

Since we had noted a significant change in the vascular function and remodelling in the A-NOS3 KO mice, we examined the surrounding PVATs to investigate the link between A-NOS3 KO and the observed vascular phenotypes. The gene expression of *Nos3* was significantly reduced in the PVAT and mPVAT of A-NOS3 KO mice compared to that of control mice (see Supplementary material online, *Figure S2*). Next, the gene expression of inflammatory cytokines was examined by qPCR. The results revealed that the expression levels of interleukin 6 (*Il-6*), intercellular adhesion molecule 1 (*Icam-1*), and vascular cell adhesion molecule 1 (*Vcam-1*) were significantly upregulated in the aortic PVAT of A-NOS3 KO HFD mice compared to control HFD mice (*Figure 5A* and Supplementary material online, *Figure S3A*), while the expression levels of tumour nuclear factor alpha (*Tnfa*), *Il-6*, monocyte chemoattractant protein-1 (*Mcp-1*), and *Vcam-1* were significantly upregulated in the mPVAT of A-NOS3 KO HFD mice compared to control HFD mice (*Figure 5B* and Supplementary material online, *Figure S3B*).

Next, we investigated the gene expressions of immune cell markers in the aortic PVAT. A-NOS3 KO mice exhibited an upregulation of B-cell activating factor (*Baff*) compared to control under both NCD and HFD conditions. At the same time the expression of other B-cell-related markers, including *CD45* and *B220* was significantly upregulated in the A-NOS3 KO HFD group compared to control HFD group (*Figure 5A* and Supplementary material online, *Figure S3C*). A-NOS3 KO HFD mice exhibited an upregulation of signature markers of T-cell, including *CD4*, *CD8*, and T-box transcription factor (*Tbx21*) compared to control HFD. At the same time, there was no significant difference in the expression of CD3-gamma unit (*CD3-GU*), chemokine (C-C motif) ligand 5 (*CCL5*), and GATA binding protein 3 (*Gata3*) genes between control and A-NOS3 KO HFD mice (*Figure 5A* and Supplementary material online, *Figure S3D*). However, there was no significant difference in the expression of markers related to monocytes/macrophages in the PVAT between control and A-NOS3 KO mice (*Figure 5A* and Supplementary material online, *Figure S3E*).

In mPVAT, A-NOS3 KO mice under both NCD and HFD conditions exhibited an upregulation of B-cell activating factor (*Baff*) compared to control groups, while the expression of *CD45* was significantly upregulated in the A-NOS3 KO HFD compared to control HFD and A-NOS3-KO NCD (*Figure 5B* and Supplementary material online, *Figure S3F*). In the mPVAT of A-NOS3 KO HFD mice, the expression of signature markers of T-cell including *CD4*, *CD8*, *CCL5*, *Tbx21*, and *Gata3* was significantly upregulated compared to that in control HFD (*Figure 5B* and Supplementary material online, *Figure S3G*). A-NOS3 KO HFD mice exhibited an upregulation in the signature markers of monocytes/macrophages, including *CD11b*, *CD68*, epidermal growth factor -like module-containing mucin-like hormone receptor-like 1 (*Emr1*), as well as markers of M1 macrophages (*CD11c* and *CCL2*), compared to control HFD. There was no significant difference in the expression of markers of M2 macrophages in the mPVAT between control and A-NOS3 KO HFD mice (*Figure 5B* and Supplementary material online, *Figure S3H*).

Hypoxia is a major trigger for adipose tissue dysfunctions, including adipocyte remodelling, inflammation, reactive oxygen species generation, and oxidative stress.^31^ Therefore, we examined the expression of genes related to hypoxia in the PVAT and mPVAT of our experimental models. Strikingly, hypoxia-inducible factor 1-alpha (*Hif-1a*), the signature marker of hypoxia, and its downstream target vascular endothelial growth factor (*Vegf*) and ten-eleven translocation-1 (*Tet1*) were significantly upregulated in both the aortic PVAT and mPVAT of A-NOS3 KO HFD mice compared to that of control HFD (*Figure 5* and Supplementary material online, *Figure S3I* and *J*). Various oxidative stress markers, including *Nox2* and *Nox4*, were also upregulated in the PVAT and mPVAT of A-NOS3 mice compared to that of control mice after HFD feeding (see Supplementary material online, *Figure S4A* and *B*).

### 3.6 Chemerin expression is upregulated in adipocyte-specific NOS3 KO mice

**Figure 6:**
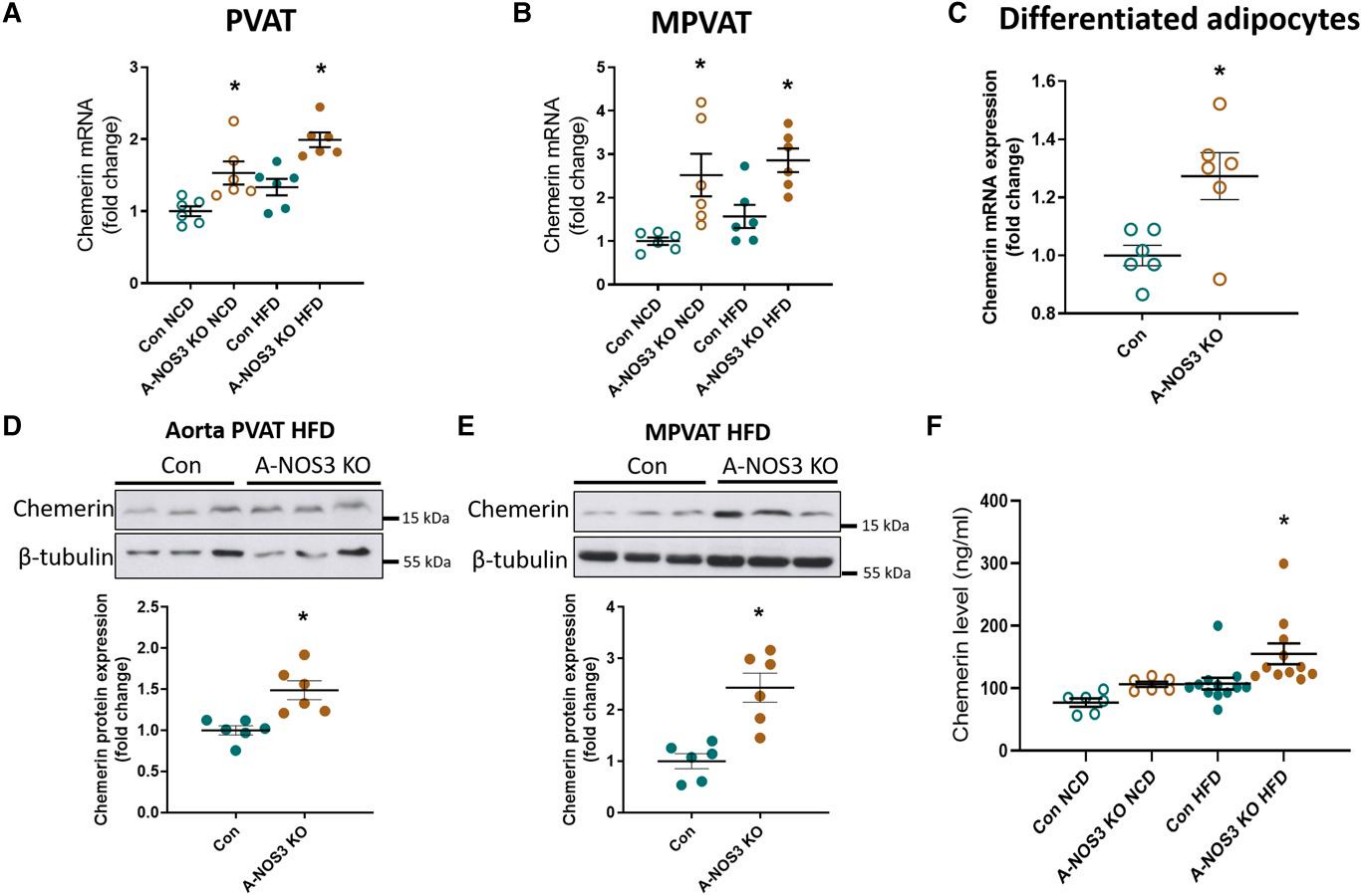
Chemerin expression is upregulated in adipocyte-specific NOS3 KO mice. (*A*) Gene expression of chemerin in aortic PVAT was analysed by quantitative PCR. (*B*) Gene expression of chemerin in mPVAT was analysed by quantitative PCR. (*C*) Gene expression of chemerin in differentiated adipocytes cultured from the SVF of white adipose tissues isolated from control and A-NOS3 KO mice was analysed by quantitative PCR. (*D*) Protein expression of chemerin was analysed in PVAT using western blotting. (*E*) Protein expression of chemerin was analysed in mPVAT using western blotting. (*F*) Serum levels of chemerin in control NCD, A-NOS3 KO NCD, control HFD and A-NOS3 KO HFD mice were measured using ELISA. One-way ANOVA followed by Tukey's *post hoc* test was used to compare multiple groups (*A, B,* and *F*). Student’s *t*-test was used to compare control and A-NOS3 KO groups (*C, D,* and *E*). *P < 0.05 vs. control of the same diet. ^#^*P* < 0.05 vs. NCD of the same genotype.

Adipose tissue inflammation can lead to adipose tissue dysfunction and adipokine dysregulation.^32^ We examined the gene expression of various adipokines in PVAT and mPVAT (see Supplementary material online, *Figure S5*). Among the differentially expressed adipokines, we noticed an adipokine of interest, chemerin, which could be involved in the observed vascular phenotypes. Chemerin, a novel adipokine, has been recently considered a regulator of adipogenesis, inflammation, and a missing link between obesity and vascular dysfunction.^23^ The gene expression of chemerin was significantly upregulated in the PVAT and mPVAT of A-NOS3 KO NCD mice and A-NOS3 KO HFD mice compared to their respective control mice (*Figure 6A* and *B*). In addition, differentiated primary adipocytes isolated from A-NOS3 KO mice exhibited a significant upregulation of chemerin mRNA expression compared to those from control mice (*Figure 6C*). Deletion of NOS3 in adipocytes led to a slight increase in chemerin protein expression in the aortic PVAT samples of HFD mice (*Figure 6D*), while its protein expression was more than doubled in the mPVAT A-NOS3 KO HFD mice compared to control HFD mice (*Figure 6E*). Moreover, A-NOS3 KO HFD mice exhibited a significantly higher serum level of chemerin than control HFD mice (*Figure 6F*).

### 3.7 Chemerin neutralization normalizes remodelling-related genes expression *ex vivo*

**Figure 7:**
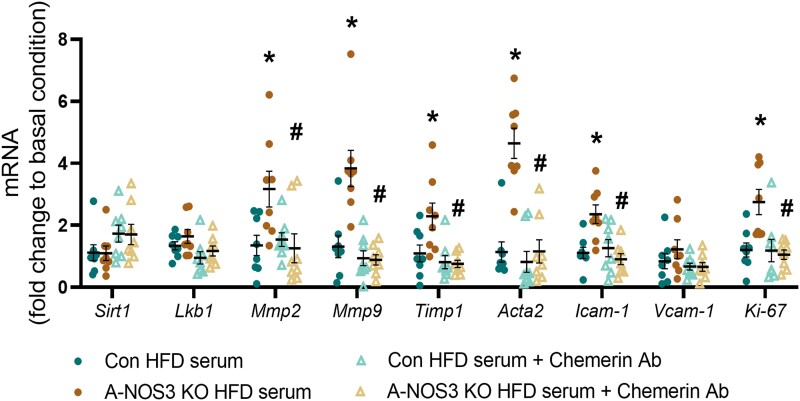
Chemerin neutralization normalizes remodelling-related genes expression *ex vivo.* Aorta from 12 weeks old control mice fed with NCD (donor mice) was isolated and PVAT was removed from the aorta. Aorta segments were cultured in basal Dulbecco's modified Eagle's medium (DMEM) in the presence of 15% serum of either the donor mice itself, serum of control HFD group or A-NOS3 KO HFD group. Aorta segments were incubated additionally with either goat IgG (10 µg/mL) or chemerin neutralizing antibody (10 µg/mL). The aorta segments were incubated for 48 h. Gene expression of Sirtuin 1 (Sirt1), liver kinase B1 (Lkb1), and signature markers of remodelling including matrix metalloproteinase 2 and 9 (*Mmp2* and *Mmp9*), metallopeptidase inhibitor 1 (*Timp1*), smooth muscle alpha actin (*Acta2*), intercellular adhesion molecule 1 (*Icam-1*), and vascular cell adhesion molecule 1 (*Vcam-1*), and *Ki-67* in the cultured aorta segments was analysed by quantitative PCR. qPCR results of this *ex vivo* aorta culture experiment were normalized against the group cultured with the serum of donor mice. One-way ANOVA followed by Tukey's *post hoc* test was used to compare multiple groups. *P < 0.05 vs. control HFD group. ^#^*P* < 0.05 vs. A-NOS3 KO HFD group.

Since the serum level of chemerin was significantly higher in A-NOS3 KO HFD mice compared to control HFD mice, we utilized those serum samples to culture the aorta isolated from control mice *ex vivo*, with chemerin being neutralized by antibody binding. In the aorta segments cultured with A-NOS3 KO HFD serum, there was a significant upregulation of signature markers of vascular remodelling, including *Mmp2*, *Mmp9*, *Timp1*, *Acta2*, *Icam-1*, and *Ki-67*, compared to those cultured with control HFD serum (*Figure 7*). Incubation with either control HFD or A-NOS3 KO HFD serum had no significant effects on the expression levels of genes related to Nox and antioxidant enzymes (see Supplementary material online, *Figure S6A* and *B*), very likely due to the absence of immune cell infiltration in this experimental setup. Strikingly, chemerin neutralization by antibody normalized the expression of signature markers of vascular remodelling in the aorta cultured with A-NOS3 KO HFD serum (*Figure 7*). At the same time, chemerin antibody did not cause any significant changes in the expression of genes related to Nox and antioxidant enzymes (see Supplementary material online, *Figure S6A* and *B*).

## 4. Discussions

In the present study, we investigated the impact of adipocyte-specific knockout of NOS3 on diet-induced hypertension and its associated vascular dysfunction and remodelling. HFD feeding in A-NOS3 KO mice resulted in (i) an exaggerated blood pressure elevation, (ii) endothelial and PVAT dysfunction that was characterized by the reduced acetylcholine-evoked vascular relaxation and the reduced anti-contractile function of the surrounding PVAT, (iii) vascular remodelling characterized by the elevated vascular smooth muscle proliferation and collagen deposition in the aorta, (iv) elevated inflammation and hypoxia in the aortic PVAT and mPVAT, and (v) the upregulation of chemerin, a novel adipokine involved in metabolic disorders. This is the first study to utilize a tamoxifen-induced adipocyte-specific knockout mouse model to evaluate the phenotype of NOS3 knockout in adipocytes and its roles in vascular function. Moreover, we demonstrate that the neutralization of chemerin could normalize the expression of vascular remodelling markers *ex vivo*. These data show that deleting adipose NOS3 potentiates HFD-induced hypertension and vascular remodelling, at least partly, via chemerin signalling.

In recent years, expression of NOS3 in cell types other than endothelial cells has been demonstrated *in vitro* and *in vivo.*^33^ Adipose tissue is a conglomerate of various cell types, including adipocytes, preadipocytes, immune cells, and mesenchymal stem cells, and is invested with a network of microvessels.^34^ Coherent with other studies, we have previously demonstrated the presence of NOS3 in the adipocytes in mouse PVAT.^16^ In our A-NOS3 KO mice, NOS3 is only deleted in the adipocytes but not in the SVF of adipose tissues (*Figure 1E*). Until now, most of the knowledge about adipose NOS3 is based on evidence from studies using global eNOS knockout mice or mice with pathological conditions that lead to the downregulation of NOS3 in adipose tissues. Therefore, our A-NOS3 KO model can be used as a tool for investigating the specific function of adipocyte NOS3 in metabolic and vascular homeostasis. Strikingly, the deletion of adipocyte NOS3 led to an exaggeration of HFD-induced hypertension in the present study.

Endothelial dysfunction is an important risk factor for the development of arterial hypertension. Endothelial dysfunction not only leads to functional deterioration and impaired control of the vascular tone but also gradually stimulates the structural changes of the blood vessels, such as intimal thickening of the vessel wall and collagen deposition.^35^ Indeed, obese individuals exhibit blunted vasodilatation in response to acetylcholine in resistance arteries and reduced capillary recruitment in response to reactive hyperaemia and shear stress,^36,37^ suggesting the association between endothelial dysfunction and obesity-induced hypertension. Moreover, the correlation between the severity of endothelial dysfunction and the degree of adiposity has been documented since 1999, although the degree of vascular dysfunction can be independent of body weight.^37^ Despite the association between obesity, hypertension, and vascular dysfunction, the underlying complex mechanisms remain controversial. The present study provides direct evidence that NOS3 in adipocytes may be an essential link between adipose tissue and blood pressure regulation. Deleting adipocyte NOS3 potentiates HFD-induced blood pressure increases in mice despite a reduced weight gain (*Figure 2*).

Moreover, we have shown that the deletion of adipocyte NOS3 led to an impairment of endothelial and PVAT functions in the A-NOS3 KO mice, even under normal diet feeding and in vessel ring without PVAT, while the endothelial and PVAT dysfunctions were worsened upon HFD feeding (*Figure 3*). These findings suggest an important cross-talk between adipose tissue (particularly the PVATs) and the vascular wall, with a significant contribution from the NOS3 in adipocytes. The resulting changes in the vascular wall leads to a permanent impairment of vascular function, which was also evident even when the PVAT was removed in *ex vivo* experiments (*Figure 3*).

Indeed, recent studies have demonstrated the potential role of PVAT in the vascular dysfunction occurring in obesity.^38,39^ PVAT is crucial in vascular NO production.^16,18,22^ It has been demonstrated that, under healthy conditions, PVAT can attenuate the vascular responsiveness to several constrictor agonists, while it can also release relaxation factors.^38,40^ The endothelium-independent anti-contractile function of PVAT has also been demonstrated.^41,42^ In experimental models of obesity, the ability of PVAT to release relaxation factors is impaired.^43^ Indeed, the endothelium-dependent NO-mediated acetylcholine-induced vasodilation response is not changed in PVAT-removed aortas from HFD-fed wildtype mice compared with NCD wildtype mice, while vascular dysfunction of the thoracic aorta is only evident when PVAT is adhered.^16,25^ These results suggest that the effect of obesity in vascular dysfunction could occur from PVAT. It has been reported that PVAT mediates the vascular function and remodelling in HFD-induced obesity, which is associated with impaired NOS3-mediated signalling.^44^ In our A-NOS3 KO mice model, our results coherently demonstrated that the pathology of vascular dysfunction and remodelling was enhanced in the absence of adipocyte NOS3 (*Figures 3* and *4*), which was associated with augmented expression of oxidative stress markers in the vessel wall. Vascular remodelling is an active process that involves multiple levels, including vascular smooth muscle cells (VSMC) proliferation, migration, cell death, and extracellular matrix remodelling, which can be evoked by various growth factors, inflammatory cytokines, and vasoactive substances.^45,46^ Vascular dysfunction and remodelling associated with obesity trigger functional alterations and can lead to impaired tissue perfusion that may affect multiple tissues and organs. Coherent with our results, the proliferation of VSMCs is a common characteristic reported in the vessels in the context of obesity.^47^

Interestingly, endothelium-dependent vasodilation of both the aorta and the mesenteric artery was reduced in A-NOS3 KO NCD mice compared to control NCD mice (*Figure 3*), but without an elevation of the blood pressure in the NCD mice (*Figure 2*). Previous studies have shown that endothelial dysfunction may precede the development of hypertension.^48^ In the rat model of diet-induced insulin resistance, impairment of endothelium-mediated relaxation is evident before the onset of blood pressure elevation.^49^ Normotensive postmenopausal women with impaired endothelial function have an >5-fold increased risk of developing hypertension.^50^ Similarly, acetylcholine-induced forearm vasodilation is reduced in normotensive individuals with a familial history of essential hypertension, indicating that endothelium dysfunction can precede the appearance of hypertension.^51^ In our study, A-NOS3 KO mice on NCD showed reduced endothelium-dependent vasodilation without increased blood pressure. Compared to A-NOS3 KO mice on HFD, only little vascular remodelling was observed in A-NOS3 KO NCD mice (*Figure 4*). Therefore, we postulate that an impairment of endothelial dysfunction alone is insufficient to induce hypertension in our experimental setting. Instead, remodelling and stiffening of the vascular wall play a decisive role in the development of hypertension in this model.

Moreover, the absence of adipocyte NOS3 resulted in an exaggeration of inflammation and hypoxia in aortic PVAT and mPVAT of A-NOS3 KO mice fed with HFD (*Figure 5*). The detailed mechanisms on how PVAT contributes to vascular remodelling are currently unclear. Nevertheless, the transplantation of PVAT from HFD-fed mice on the carotid artery accelerated vascular remodelling after a wire-induced injury in low-density lipoprotein receptor knockout mice.^52^ These authors have demonstrated that monocyte chemoattractant protein-1 (MCP-1) in PVAT is, at least partly, involved in the PVAT-induced vascular remodelling in obesity. In a very recent study, inhibition of PVAT beiging has been demonstrated to exacerbate PVAT inflammation and vascular remodelling following injury. In contrast, activation of PVAT beiging attenuates PVAT inflammation and pathological vascular remodelling,^53^ suggesting that the inflammatory status of PVAT may play a crucial role in the progress of pathological vascular remodelling.

Chemerin is a novel adipokine first identified as a molecule promoting the chemotaxis of immature dendritic cells and macrophages.^54^ Since then, chemerin has been associated with chronic inflammation and many other diseases, including cardiovascular diseases and cancers.^55,56^ In human studies, chemerin gene expression and circulating levels correlate positively with increased body mass index and obesity-related biomarkers,^57^ while patients with hypertension have significantly higher levels of serum chemerin.^58,59^ In mice, plasma chemerin levels are augmented in diet-induced obesity and reduced by overnight fasting.^60^ Others have reported elevated levels of circulating chemerin in inflammatory states.^61^ Chemerin is expressed at high levels in white adipose tissues but only in low levels in BATs,^62^ and its expression is upregulated in adipocytes of mice fed with HFD.^63^ Despite the association between chemerin level, hypertension, and obesity, the regulation mechanisms of chemerin expression are not clear. In this present study, we have revealed that the serum level of chemerin and the expression of chemerin in the PVATs were augmented in A-NOS3 KO mice fed with HFD (*Figure 6*), suggesting that NOS3 in adipocytes may play a crucial role in regulating chemerin expression in adipose tissues.

A high level of chemerin has recently been associated with the upregulation of VEGF and angiogenesis.^64,65^ In obesity, chemerin may mediate the abnormal vascular smooth muscle contractility and promote VSMC proliferation.^66,67^ In a recent study, chemerin exacerbates pulmonary arterial hypertension by promoting the proliferation and migration of human pulmonary artery smooth muscle cells.^68^ In support to these studies, our *ex vivo* aorta culture experiment suggested that the high serum level of chemerin in A-NOS3 KO HFD mice may trigger the pathological vascular remodelling process, evidenced by the direct effect in upregulating remodelling markers. In contrast, the neutralization of chemerin could normalize such changes (*Figure 7*). Indeed, chemerin has been reported to stimulate the expression of MMP-2 and MMP-9.^65^ Together, these findings suggest that the vascular dysfunction and remodelling observed A-NOS3 KO mice could be, at least partly, attributed to the high serum level of chemerin.

The current understanding of eNOS/NOS3 functions in adipose tissue is highly based on findings concerning eNOS function in endothelial cells and experiments conducted using global knockout mice or animal models with pathological conditions. The strength of our present study is the use of adipocyte-specific NOS3 knockout mice. This is the first study that reports the phenotype and pathology of adipocyte-specific NOS3 knockout mice fed with HFD. Our results demonstrate the effect of adipocyte-specific NOS3 knockout in potentiation of HFD-induced hypertension, which directly supports the importance of NOS3 in adipocytes. Future clinical studies should focus on the expression and function of NOS3 in adipose tissues of patients with obesity and/or related diseases.

Nevertheless, our study has certain limitations. First, A-NOS3 KO mice gained less body weight on HFD than the control mice. The mechanisms underlying this phenomenon are beyond the scope of the present study and are currently being addressed separately. Nevertheless, the fact that A-NOS3 KO mice have higher blood pressure despite less weight gain further underlines the importance of adipocyte NOS3 in diet-induced hypertension. Second, large and small arteries differ in their vasomotion mechanisms and may play different roles in obesity-induced hypertension.^69,70^ We studied vascular function of both the aorta and mesenteric arteries, while we only focused on the vascular remodelling in the aorta but not in the mesenteric arteries. Obesity is also associated with coronary microvascular dysfunction,^69,70^ but this could not be addressed in the present study. Third, we did not investigate the detailed molecular mechanisms on how adipose NOS3 contributes to the regulation of chemerin expression. This is an important yet complex question that needs to be addressed in future studies. Fourth, mitochondria are involved in both adipose tissue function and vascular function.^71^ Ablation of NOS3 may affect the mitochondrial function and lead to adipose tissue and vascular dysfunction, which was not analysed in our study. Fifth, we observed a normalization of remodelling marker expression in the aorta by *ex vivo* chemerin neutralization, indicating a direct effect of chemerin in regulating vascular remodelling. This is consistent with previous findings that chemerin inhibition can normalize the VSMC dysfunction *in vitro.*^72^ However, chemerin neutralization did not affect the expression of oxidative stress markers (see Supplementary material online, *Figure S6*), which could be due to the absence of immune cells in our *ex vivo* model. In the absence of immune cells, no upregulation of NADPH oxidase components was observed in the aorta from healthy control mice incubated *ex vivo* with serum from A-NOS3 KO HFD mice (see Supplementary material online, *Figure S6*), which contrasts with the increased expression of Nox2, p22phox, and p47phox in the aorta of A-NOS3 KO HFD mice (see Supplementary material online, *Figure S1).* This discrepancy supports the idea that the oxidative stress in the vascular wall of A-NOS3 KO HFD mice *in vivo* originates mainly from infiltrated immune cells, as a response to the pro-inflammatory cytokine production from the PVAT. Sixth, we only looked at the effect of chemerin neutralization at mRNA level, while further investigation in the function and morphology is warranted. Moreover, demonstrating the causal role of chemerin in obesity-induced hypertension requires further studies and *in vivo* inhibition of chemerin levels in A-NOS3 KO mice is needed to confirm the pathological role of the augmented chemerin level. Seventh, the NOS3 gene displays single nucleotide polymorphisms associated with lower NOS3 activity and endothelial dysfunction.^73^ It is conceivable that NOS3 polymorphisms also affect NOS3 function in adipocytes. This, however, remains to be shown and needs to be addressed in future studies. Finally, our study did not include a Cre-expressing control group. In some models, Cre expression itself (in the absence of LoxP) can result in toxicity.^74,75^ Including a group of tamoxifen-treated Adipoq-iCreERT2 mice that lack floxed NOS3 gene may control the potential Cre toxicity. On the other hand, the potential Cre toxicity in the Adipoq-iCreERT2 mice has been extensively examined previously. Compared with the tamoxifen-treated littermate Cre-negative control mice, tamoxifen-treated Adipoq-iCreERT2 mice had normal body weight development. Moreover, these animals showed no abnormalities in glucose tolerance, insulin sensitivity, lipolysis, O_2_ consumption, CO_2_ production, respiratory quotient, feeding behaviour, or activity.^24^ Thus, in the Cre mouse line used in our study, Cre toxicity has not been observed. In addition, tamoxifen injection had no adverse effects on the body weight development of the animals (see Supplementary material online, *Figure S7*).

In summary, we examined the phenotype of a novel mouse model for adipocyte-specific gene knockout of eNOS/NOS3. The comparison of mice with or without adipocyte NOS3, especially when investigating adipocytes, directly demonstrates the critical role of adipocyte NOS3. For the first time, we present direct evidence demonstrating that the absence of adipocyte NOS3 leads to the exaggeration of diet-induced hypertension, which is associated with vascular dysfunction and remodelling. The pathological vascular phenotype can be attributed to the inflammatory status of the surrounding PVATs. Interestingly, we have also revealed that a novel adipokine, chemerin, is upregulated in A-NOS3 KO mice. Chemerin is likely the molecular link between the absence of adipocyte NOS3 and vascular dysfunction and remodelling, as evidenced by the *ex vivo* neutralization experiment. The data presented herein, and the usage of our mice model, provide a better understanding of the specific role that adipocyte eNOS plays in normal physiology and obesity-related complications. NOS3-chemerin pathway may act as an important link between adipose tissues and the vasculature.

## Supplementary material

Supplementary material is available at *Cardiovascular Research* online.

## Supplementary Material

cvad164_Supplementary_Data

## Data Availability

The datasets used and/or analysed during the current study are available from the corresponding authors on reasonable request.

## Notes

Translational perspective The role of NOS3 in adipocytes has been rarely investigated so far. This study demonstrates that the absence of adipocyte NOS3 potentiates diet-induced hypertension and reveals chemerin as a molecular link between adipose tissues and the vasculature. These findings suggest the possibility of pharmacological therapies to treat metabolic diseases by targeting adipocyte NOS3 and/or chemerin and to improve cardiovascular health.
