# Supplementary material for: Deletion of adipocyte NOS3 potentiates high-fat diet-induced hypertension and vascular remodelling via chemerin
Source: Cardiovasc Res. 2023 Oct 28;119(17):2755–69. doi: 10.1093/cvr/cvad164 (PMC10757584; doi:10.1093/cvr/cvad164)
Supplement: cvad164_Supplementary_Data [file cvad164_supplementary_data.pdf]

Supplementary table S1. Primer used in this study

Gene	Forward	Reverse
Acta2	TTCGTGTGGCCCCTGAAGAGCAT	CCAGTTGTACGTCCAGAGGCA
Adiponectin	GTTGCAAGCTCTCCTGTTCC	ATCCAACCTGCACAAGTTCC
Apelin	TGAATCTGAGGCTCTGCGTG	ACATCAGTGGCACTCCACAA
B220	CCATGGGTTTGTGGCTCAA	TGCCCTGTGACAAAGACTTCTG
b-actin	CCTCTATGCCAACACAGTGC	CACACAGAGTACTTGCGCTC
Baff	GGCAGGTACTACGACCATCTC	TGGGCCTTTTCTCACAGAAGT
CCL2	ATCCCAATGAGTAGGCTGGAGAGC	CAGAAGTGCTTGAGGTGGTTGTG
CCL5	TGCTCCAATCTTGCAGTCGT	GCGTATACAGGGTCAGAATCAAG
CD11b	AAACCACAGTCCCGCAGAGA	GGGCTTCAAAGTTGTCCACTTG
CD11c	ATTTCTGAGAGCCCAGACGA	CCATTTGCTTCCTCCAACAT
CD19	GAGAGGCACGTGAAGGTCATTG	CATGGCTCTGAGCTCCAGTATC
CD206	ATGCCAAGTGGGAAAATCTG	TGTAGCAGTGGCCTGCATAG
CD36	TGAATGGTTGAGACCCCGTG	AAGGCAGCAACTTCTAGAACAG
CD38	AAGGAGCTTCCAGTAACGCAT	ATGGGTGCTCAGGGTTCTTC
CD3-GU	CGTCCGCCATCTTGGTAGAGAGAGCAT	CTACTGCTGTCAGCTCCACCTCCAC
CD4	GGTGGAGTTGTGGGTGTTCAA	CAGGCTCTGCCCTTGCA
CD45	CTCACAACTCTTACACCATCCACTCT	GCAGCACATGTTTGCTTCGT
CD68	CTTCCCACAGGCAGCACAG	AATGATGAGAGGCAGCAAGAGG
CD8	CAAGAAGACTACCCTGAAGATGAAGA	TCAGGCCCTTCTGGGTCTCT
Chemerin	GCCCCAAGAAGGACTGGAAA	CTGAGGATCCTGCTGAGGC
Col4a1	AGCTGCCTGCGTAAGTTCAG	CAAACCGCACACCTGCTAA
Emr1	TGCATCTAGCAATGGACAGC	GCCTCCTGGATCCATTTGAA
Gata3	CCTACCGGGTTCGGATGTAA	CACACACTCCCTGCCTTCTGT
Gpx1	CCTTGCCAACACCCAGTGA	CCGGAGACCAAATGATGTACTTG
Hif-1a	TCATCAGTTGCCACTTCCCCAC	CCGTCATCTGTTAGCACCATCAC
Ho-1	GGTGATGCTGACAGAGGAACAC	TAGCAGGCCTCTGACGAAGTG
Icam-1	ACCCCGCAGGTCCAATTC	CCAGAGCGGCAGAGCAAA
Il-1b	AAGGAGAACCAAGCAACGACAAAA	TGGGGAACTCTGCAGACTCAAACT
Il-6	ACAACCACGGCCTTCCCTACTT	CACGATTTCCCAGAGAACATGTG
Ki-67	CAAAGACCCCTGGCAAAAAG	GGTTTGGTGTTTCAAAGTGTTCCT
Leptin	AGAAGATCCCAGGGAGGAAA	TGATGAGGGTTTTGGTGTCA
Lkb1	GACTTCACAGTGCCTGGACA	CACAAACAGCCTTGGGCAAA
Mcp-1	CAGAAGTGCTTGAGGTGGTTGTG	ATCCCAATGAGTAGGCTGGAGAGC
Mmp2	GCCCCCATGAAGCCTTGTTT	GGTCATAGTCCTCGGTGGTG
Mmp9	AAAGACCTGAAAACCTCCAACCT	GCCCGGGTGTAACCATAGC
Nampt	TTCCCGAGGGCTCTGTCA	GTAGCACTCTGGGTCTGTGTTTTC
Nos3	AAGCCCGGGACTTCATCAAT	CCAAACACCAGCTCGCTCTC
Nox1	GGAGGAATTAGGCAAAATGGATT	GCTGCATGACCAGCAATGTT
Nox2	CCAACTGGGATAACGAGTTCA	GAGAGTTTCAGCCAAGGCTTC
Nox4	TGTAACAGAGGGAAAACAGTTGGA	GTTCCGGTTACTCAAACTATGAAGAGT
Omentin-1	CTGGTGCAGAGGGAAGACCA	ACCTCTGGTAGCAACCATGATAA
P22phox	GGAGCGATGTGGACAGAAGTA	GCACCGACAACAGGAAGTG
P47phox	AGAGCACGGATGGCACAAAG	CCGCGGGCTGTGGTT
P67phox	GGCTTGGCCTCCCCTACA	CCGGTAGCTCAGTTTAGTGTGTTC
Resistin	AGCTGTGGGACAGGAGCTAA	AGGAAAAGGAGGGGAAATGA
Sirt1	GCCAAACTTTGTTGTAACCCTGTA	TGGTGGCAACTCTGATAAATGAA
Sod1	CCAGTGCAGGACCTCATTTTAAT	TCTCCAACATGCCTCTCTTCATC
Sod2	GCTCTGGCCAAGGGAGATG	TGTCCCCCACCATTGAACTT
Sod3	TTCTTGTTCTACGGCTTGCTACTG	AGCTGGACTCCCCTGGATTT

Tbx21	AGGGGGCTTCCAACAATG	AGACGTGTGTGTTAGAAGCACTG
Tet1	TCGTGGAGCATGTATTTCAACGGC	TCCACCCGGTTTTCACGTCACT
Timp1	GAGACACACCAGAGCAGATACC	GGGGAACCCATGAATTTAGCC
Tnfa	GCCTCTTCTCATTCCTGCTTG	CTGATGAGAGGGAGGCCATT
Vcam-1	GACTCCATGGCCCTCACTTG	CGCGTTTAGTGGGCTGTCTATC
Vegf	ACTTGTGTTGGGAGGAGGATGTC	AATGGGTTTGTCGTGTTTCTGG
Ym1	ATCTATGCCTTTGCTGGAATGC	TGAATGAATATCTGACGGTTCTGAG

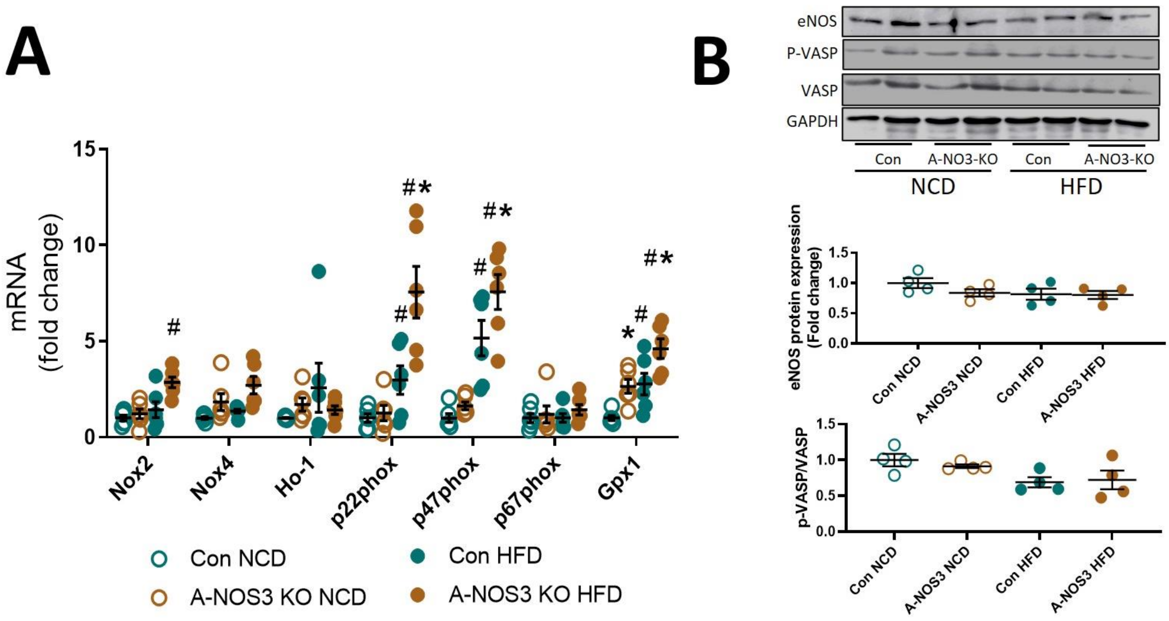

Supplementary Figure S1. Oxidative stress markers were upregulated in the aorta from A-NOS3 KO mice fed with HFD. A) The expression of oxidative stress-related genes including *Nox (2, 4)*, p*22phox*, *p47phox*, *p67phox*, heme oxygenase-1 (*Ho-1*) and glutathione peroxidase 1 (*Gpx1*) in the aorta from the four groups of mice was measured by quantitative PCR. *P<0.05 vs control of same diet. #P<0.05 vs NCD of same genotype. **B)** Total protein expression of eNOS and its downstream target vasodilator-stimulated phosphoprotein (VASP) and phosphorylated VASP (p-VASP) were measured by Western blotting in the PVAT-free aorta samples under basal condition without eNOS stimulation. GAPDH was used as reference. The ratio of phosphorylation-to-total protein was calculated. One-way ANOVA followed by Tukey's post hoc test was used to compare multiple groups. *P<0.05 vs control of the same diet. #P<0.05 vs NCD of the same genotype.

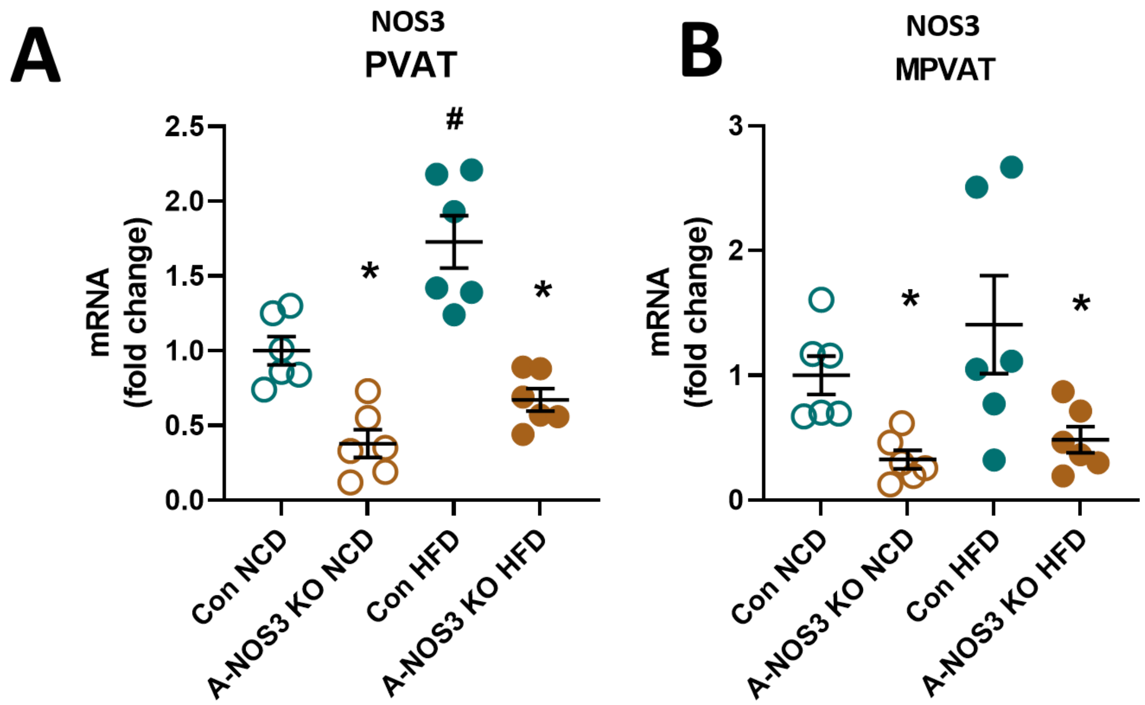

Supplementary Figure S2. NOS3 expression in the PVAT and mPVAT of the mice. The gene expression of NOS3 in the PVAT and mPVAT from the four groups of mice was measured by quantitative PCR. *P<0.05 vs control of the same diet. One-way ANOVA followed by Tukey's post hoc test was used to compare multiple groups. #P<0.05 vs NCD of the same genotype.

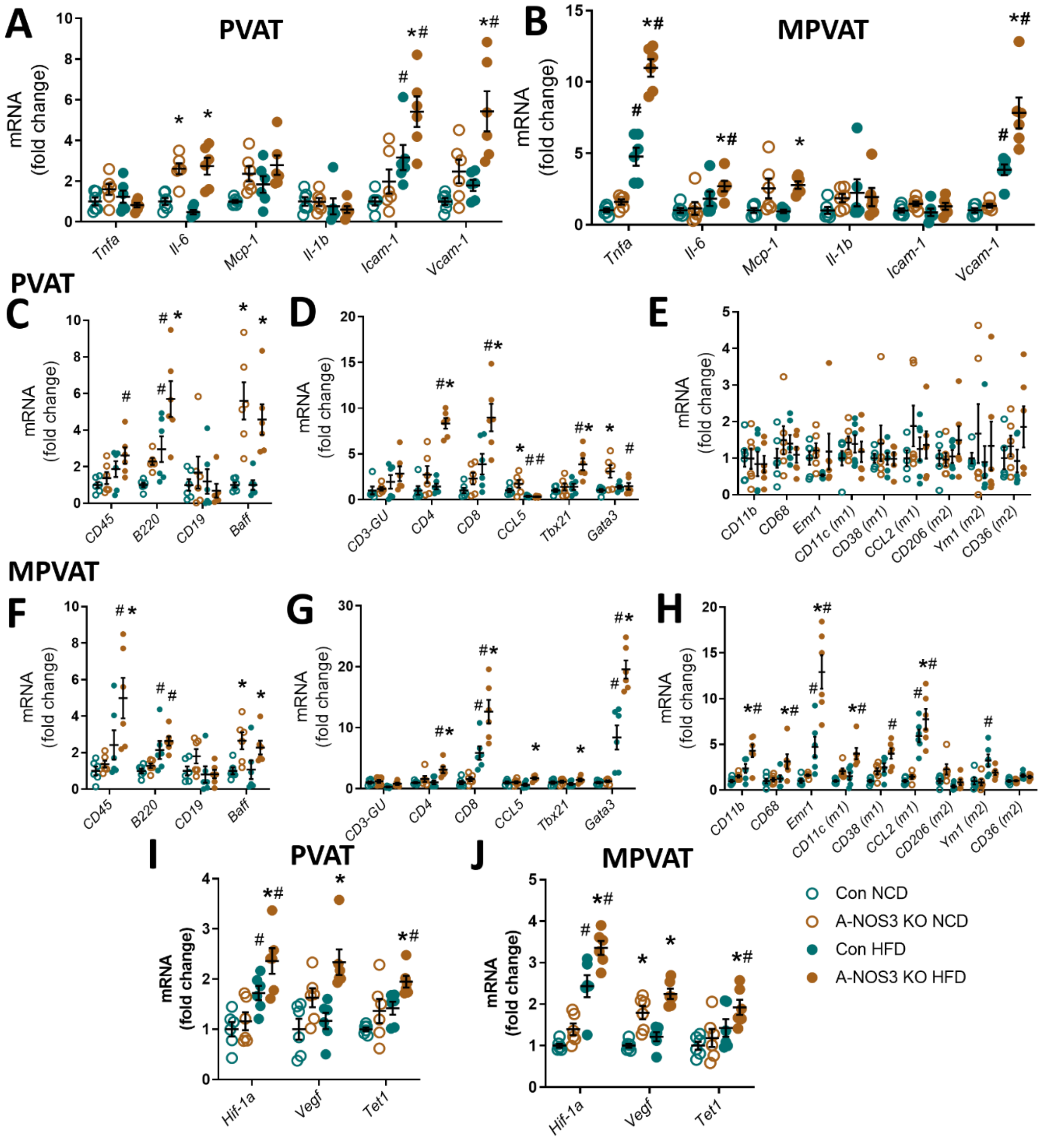

Supplementary Figure S3. Inflammation and hypoxia are augmented in HFD-fed Adipocyte-specific NOS3 KO mice. A) The expression of cytokines including tumor growth factor alpha (*Tnfa*), interleukin 6 (*Il-6*), monocyte chemoattractant protein-1 (*Mcp-1*), interleukin 1b (*Il-1b*), intercellular adhesion molecule 1 (*Icam-1*) and vascular cell adhesion molecule 1 (*Vcam-1*) in PVAT was analyzed by quantitative PCR. **B)** The expression of cytokines including *Tnfa*, *Il-6*, *Mcp-1*, *Il-1b*, *Icam-1* and *Vcam-1* in mPVAT was analyzed by quantitative PCR. **C)** The expression of B cell markers including *CD45*, *B220*, *CD19* and B-cell activating factor (*Baff*) in PVAT was analyzed by quantitative PCR. **D)** The expression of T cell markers including CD3-gamma unit (*CD3-GU*), *CD4*, *CD8,* chemokine (C-C motif) ligand 5 (*CCL5*), T-box transcription

factor (*Tbx21*) and GATA Binding Protein 3 *(Gata3)* in PVAT was analyzed by quantitative PCR. **E)** The expression of markers related to monocytes/macrophages including *CD11b*, *CD68*, EGF-like module-containing mucin-like hormone receptor-like 1 (*Emr1*), *CD11c*, *CD38*, *CCL2*, *CD206*, *Ym1* and *CD36* in PVAT was analyzed by quantitative PCR. **F)** The expression of B cell markers including *CD45*, *B220*, *CD19* and *Baff* in mPVAT was analyzed by quantitative PCR. **G)** The expression of T cell markers including *CD3-GU*, *CD4*, *CD8, CCL5*, *Tbx21* and *Gata3* in mPVAT was analyzed by quantitative PCR. **H)** The expression of markers related to monocytes/macrophages including *CD11b*, *CD68*, *Emr1*, *CD11c*, *CD38*, *CCL2*, *CD206*, *Ym1* and *CD36* in mPVAT was analyzed by quantitative PCR. **I)** The expression of markers related to hypoxia including hypoxia inducible factor 1 alpha (*Hif-1a*), vascular endothelial growth factor (*Vegf*) and ten-eleven translocation methylcytosine dioxygenase 1 (*Tet1*) in PVAT was analyzed by quantitative PCR. **J)** The expression of markers related to hypoxia including *Hif-1a*, *Vegf* and *Tet1* in PVAT was analyzed by quantitative PCR. One-way ANOVA followed by Tukey's post hoc test was used to compare multiple groups. *P<0.05 vs control of the same diet. #P<0.05 vs NCD of the same genotype.

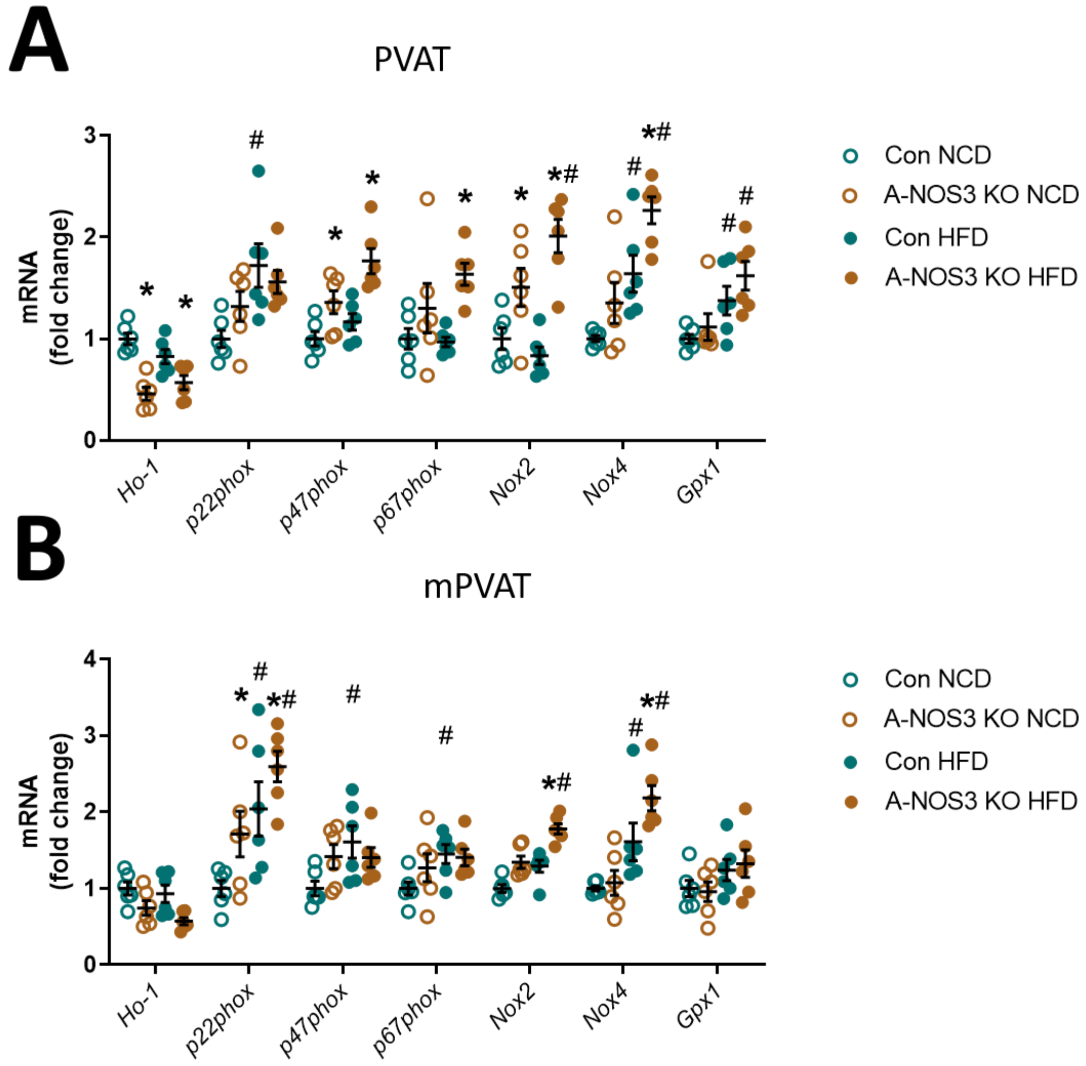

Supplementary Figure S4. Gene expression of oxidative stress markers in the PVAT and mPVAT of the mice. The gene expression of oxidative stress markers including *Nox (2, 4*), p*22phox*, *p47phox*, *p67phox*, heme oxygenase-1 (*Ho-1*) and glutathione peroxidase 1 (*Gpx1*) in the PVAT and mPVAT from the four groups of mice was measured by quantitative PCR. One-way ANOVA followed by Tukey's post hoc test was used to compare multiple groups. *P<0.05 vs control of the same diet. #P<0.05 vs NCD of the same genotype.

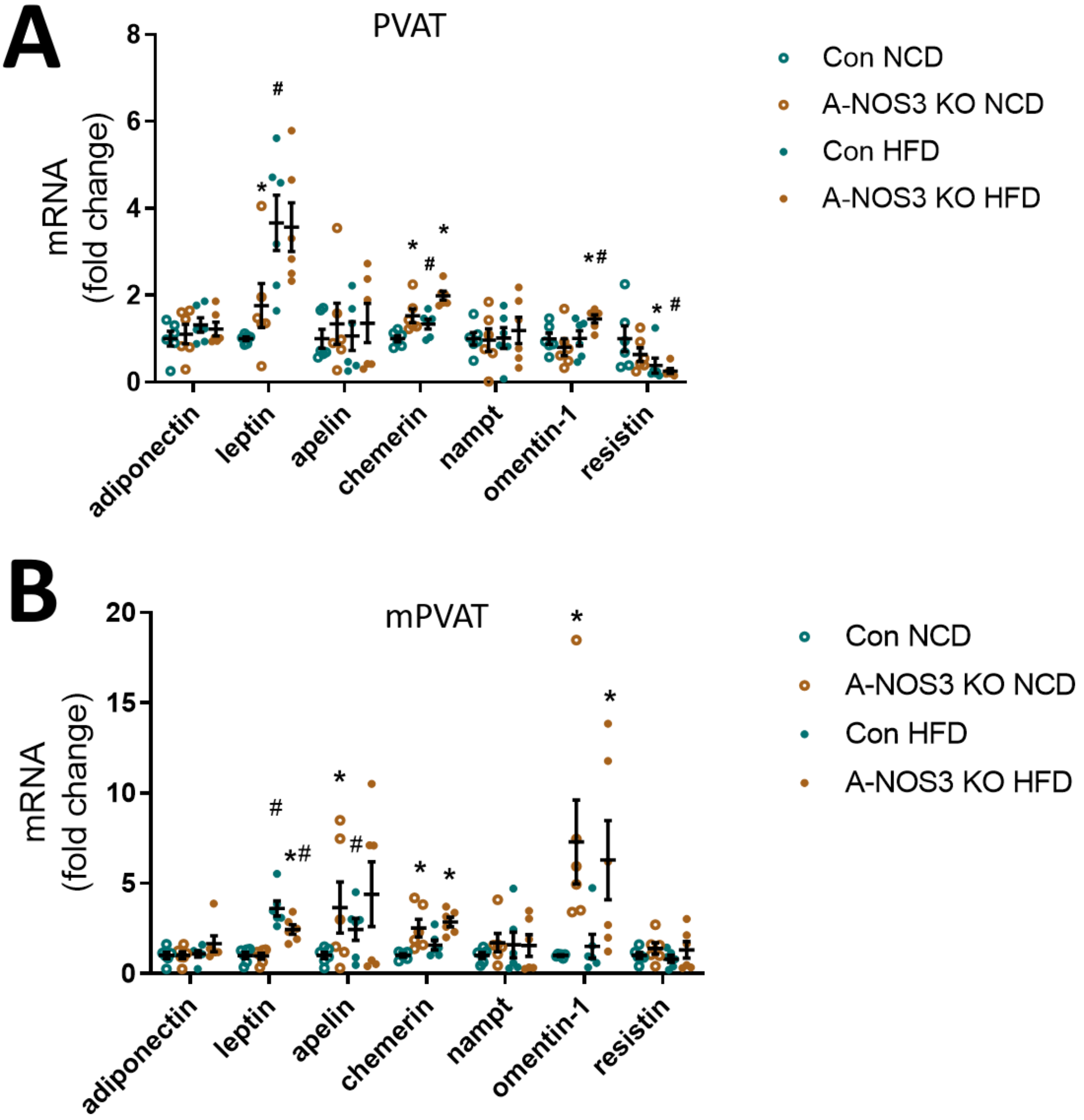

Supplementary Figure S5. Gene expression of adipokines in the PVAT and mPVAT of the mice. The gene expression of different adipokines in the PVAT and mPVAT from the four groups of mice was measured by quantitative PCR. One-way ANOVA followed by Tukey's post hoc test was used to compare multiple groups. *P<0.05 vs control of the same diet. #P<0.05 vs NCD of the same genotype.

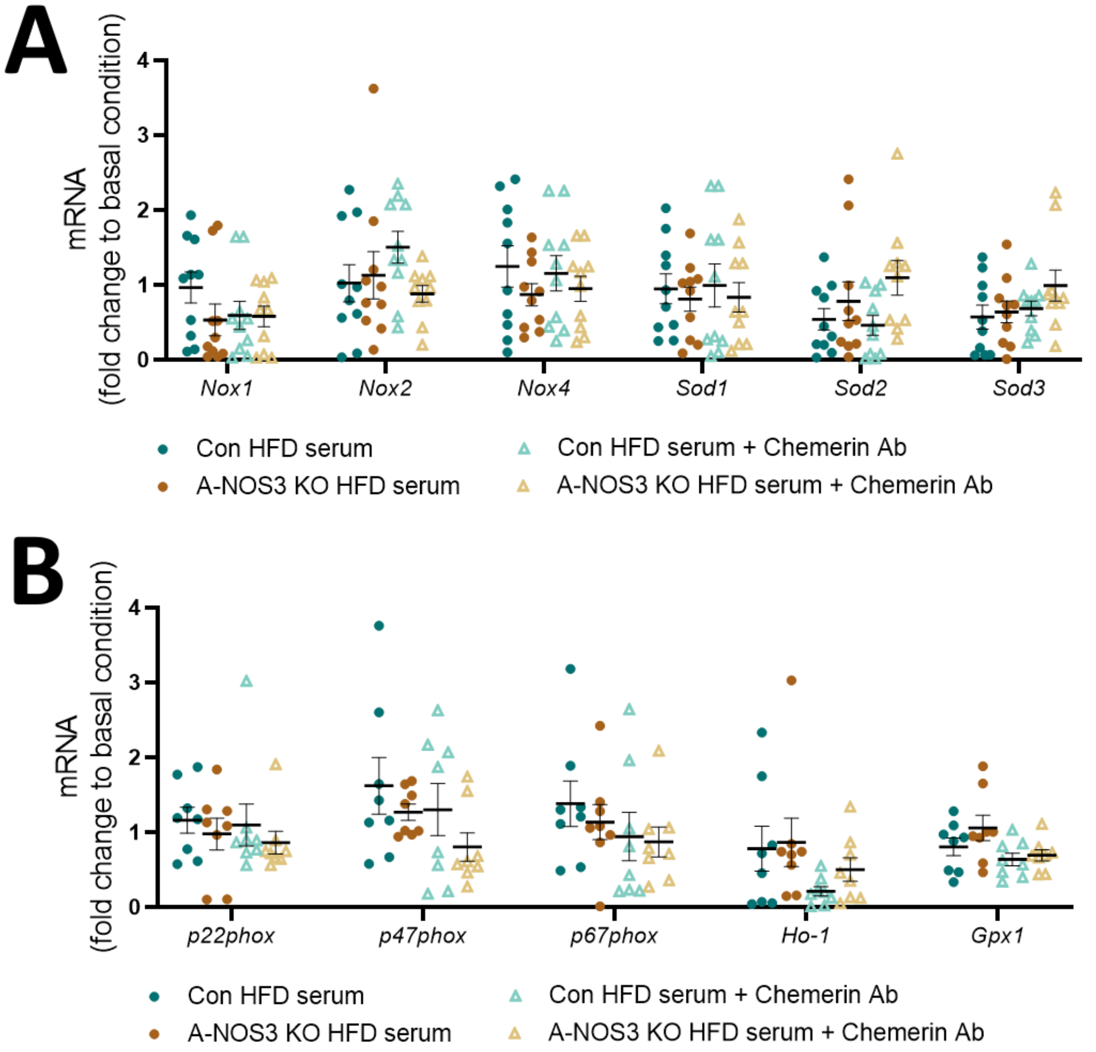

Supplementary Figure S6. ***Ex vivo*** **aorta culture had no significant changes in the expression of oxidative stress makers.** Aorta from 12-weeks-old control mice fed with NCD (donor mice) was isolated and PVAT was removed from the aorta. Aorta segments were cultured in Dulbecco's modified Eagle's medium (DMEM) in the presence of 15% serum of either the donor mice itself, serum of control HFD group or A-NOS3 KO HFD group. Some other aorta segments were incubated additionally with either goat IgG (10ug/ml) or chemerin neutralizing antibody (10ug/ml). The aorta segments were incubated for 48 hours. Gene expression of oxidative stress markers including nicotinamide adenine dinucleotide phosphate oxidases (*Nox1, 2, 4*), superoxide dismutases (*Sod1, 2, 3*), *p22phox*, *p47phox*, *p67phox*, heme oxygenase-1 (*Ho-1*) and glutathione peroxidase 1 (*Gpx1*) in the cultured aorta segments was measured by quantitative PCR. qPCR results of this *ex vivo* aorta culture experiment were normalized against the group cultured with the serum of donor mice. One-way ANOVA followed by Tukey's post hoc test was used to compare multiple groups. *P<0.05 vs control HFD group. #P<0.05 vs A-NOS3 KO HFD group.

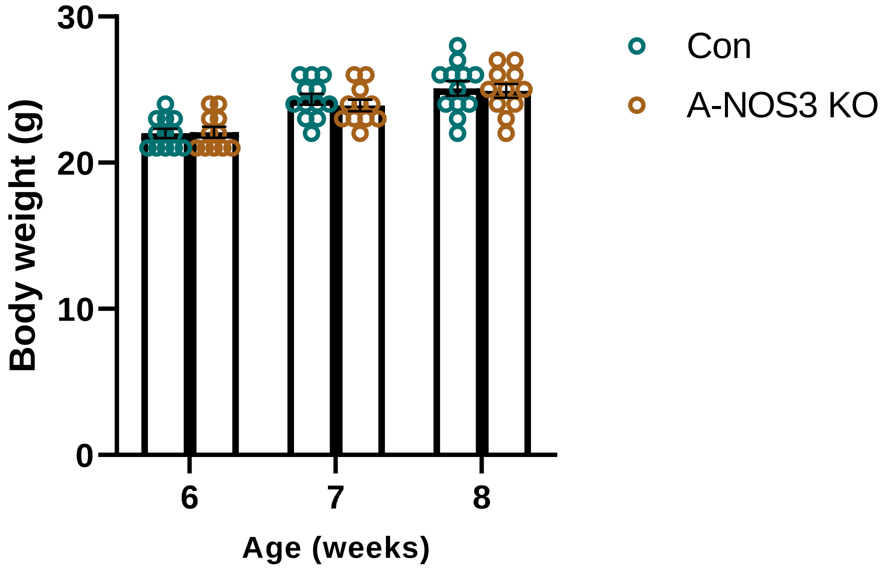

Supplementary Figure S7. Body weight of mice before and after tamoxifen injection. The body weights of the control (Con) and A-NOS3 KO mice were measured at the age of 6 weeks (before tamoxifen injection), 7 weeks (after tamoxifen injection) and 8 weeks (one week after tamoxifen injection and before diet treatment).

Figure 1 unedited gel

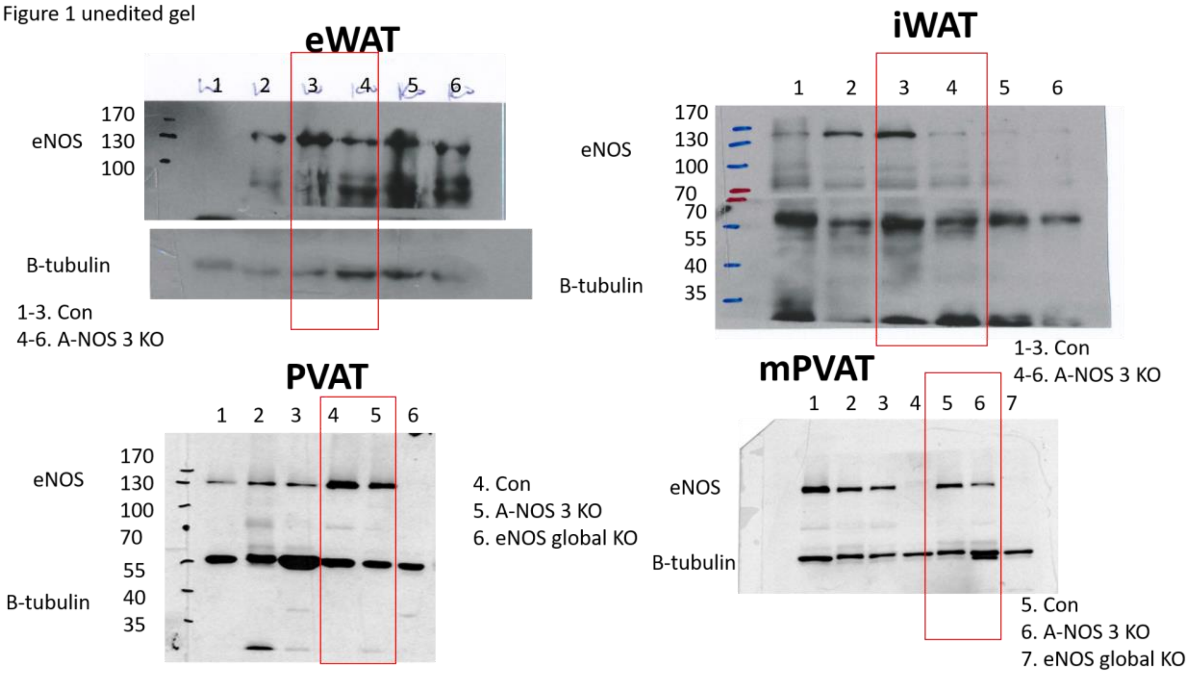

Figure 1 unedited gel

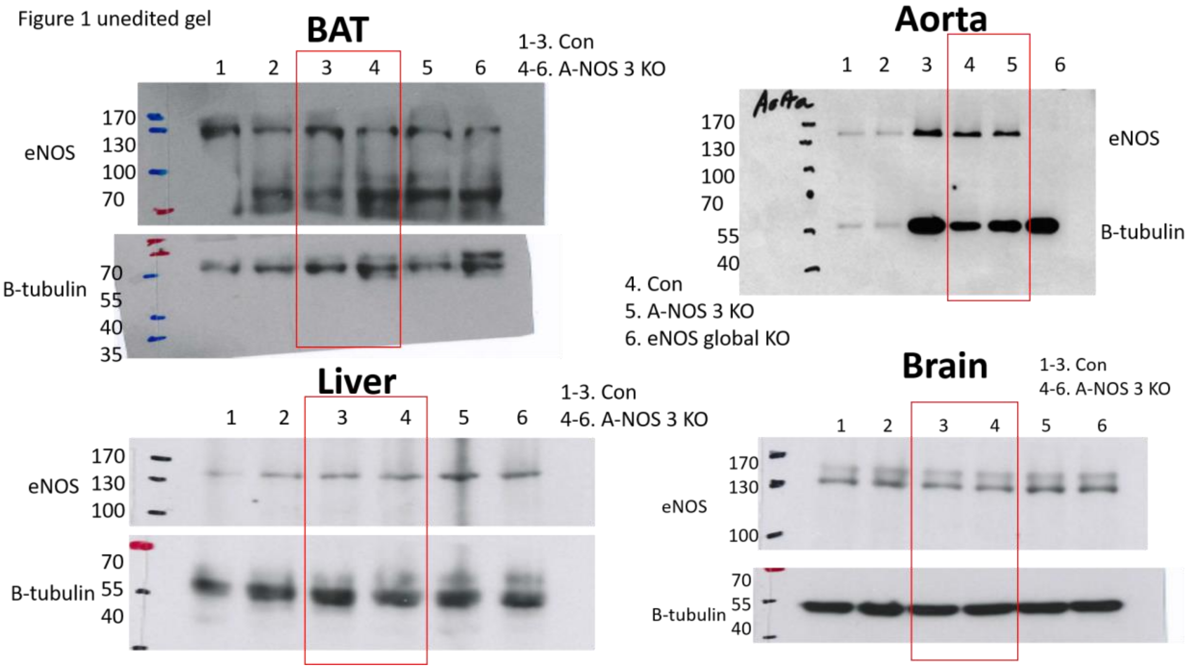

Figure 1 unedited gel

eNOS expression in adipocyte and SVF

2,3 : Control
5,6 : A-NOS3 KO

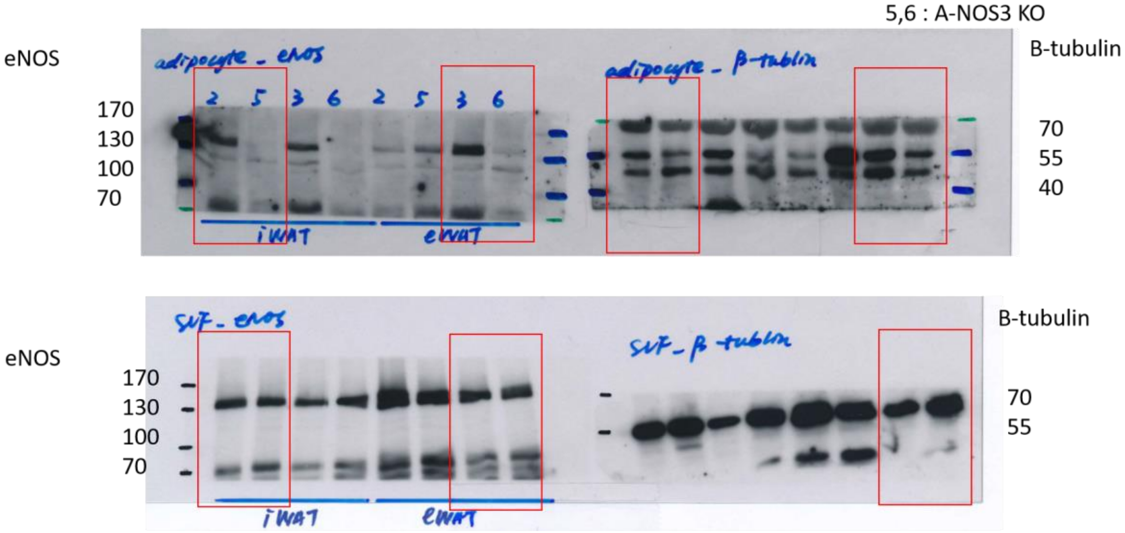

Figure 6 unedited gel

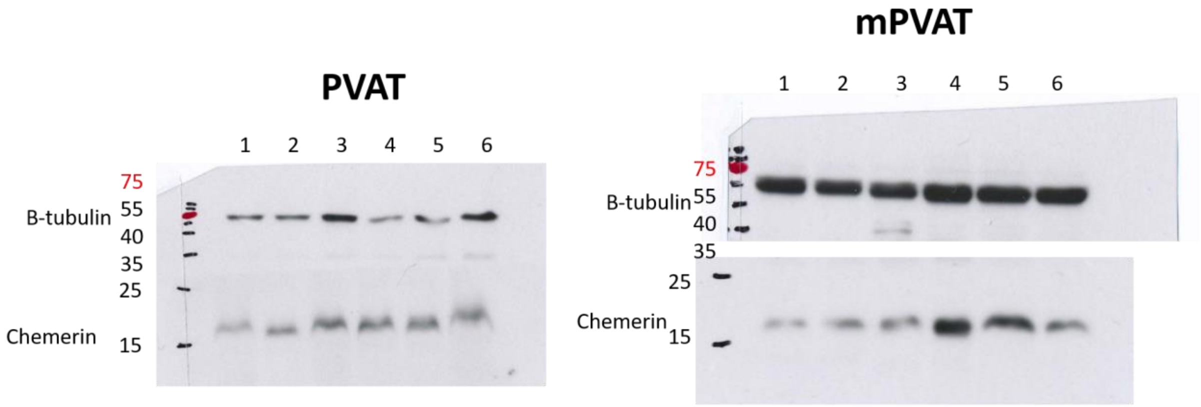

1-3. Con
4-6. A-NOS 3 KO
